# Incomplete prophage tolerance by type III-A CRISPR-Cas systems reduces the fitness of lysogenic hosts

**DOI:** 10.1038/s41467-017-02557-2

**Published:** 2018-01-04

**Authors:** Gregory W. Goldberg, Elizabeth A. McMillan, Andrew Varble, Joshua W. Modell, Poulami Samai, Wenyan Jiang, Luciano A. Marraffini

**Affiliations:** 1 Laboratory of Bacteriology, The Rockefeller University, New York, NY 10065 USA; 2 Laboratory of Systems Cancer Biology, The Rockefeller University, New York, NY 10065 USA

## Abstract

CRISPR–Cas systems offer an immune mechanism through which prokaryotic hosts can acquire heritable resistance to genetic parasites, including temperate phages. Co-transcriptional DNA and RNA targeting by type III-A CRISPR–Cas systems restricts temperate phage lytic infections while allowing lysogenic infections to be tolerated under conditions where the prophage targets are transcriptionally repressed. However, long-term consequences of this phenomenon have not been explored. Here we show that maintenance of conditionally tolerant type III-A systems can produce fitness costs within populations of *Staphylococcus aureus* lysogens. The fitness costs depend on the activity of prophage-internal promoters and type III-A Cas nucleases implicated in targeting, can be more severe in double lysogens, and are alleviated by spacer-target mismatches which do not abrogate immunity during the lytic cycle. These findings suggest that persistence of type III-A systems that target endogenous prophages could be enhanced by spacer-target mismatches, particularly among populations that are prone to polylysogenization.

## Abstract (web summary)

CRISPR-Cas systems, such as type III-A CRISPR-Cas, provide an immune mechanism for prokaryotic hosts to resist parasites, including phages. Here, the authors show that maintenance of conditionally tolerant type III-A systems can affect the fitness of *Staphylococcus aureus* lysogens.

## Introduction

Prokaryotic organisms can limit the spread of mobile genetic elements (MGEs) with the help of various defense systems, including clustered, regularly interspaced, short palindromic repeat (CRISPR) loci, and CRISPR-associated (*cas*) genes that function together as adaptive immune systems^1,2^. CRISPR–Cas systems provide immunity to invasive MGEs, such as bacteriophages (phages)^3^ and plasmids^4^, by acquiring short “spacer” sequences from their nucleic acids and incorporating them between repeat sequences of CRISPR locus DNA^5,6^. Transcription of a CRISPR locus, followed by cleavage and processing of its transcripts, generates mature CRISPR RNA (crRNA) guides containing individual spacer sequences^7^. Ultimately, crRNA-guided Cas proteins are employed to locate and degrade the nucleic acids of genetic elements that bear a matching target sequence for one or more spacer^8,9^. Four of the six (I–VI) types of CRISPR–Cas systems which have been classified to date^8,10^ include systems that cleave the DNA of their target elements^11–14^.

Given that MGEs can facilitate the spread of beneficial genes within prokaryotic populations, it was proposed that resistance to foreign DNA elements by CRISPR–Cas systems could jeopardize the survival of bacteria which rely heavily on MGE-mediated horizontal gene transfer (HGT)^4^, and in turn promote the evolution of strains that do not harbor CRISPR–Cas systems^15,16^. CRISPR–Cas systems are indeed absent from ~50% of sequenced bacterial genomes^17^, despite evidence that they can be transferred horizontally^18–21^ and function in heterologous hosts^22–24^. Moreover, the potential for genetic loss of CRISPR–Cas systems has been clearly demonstrated in laboratory settings where both beneficial plasmids and temperate phages were targeted. During the anti-plasmid CRISPR–Cas immune response, population bottlenecks imposed by antibiotics were found to select for mutant or deleted CRISPR–Cas systems when a target plasmid carrying resistance to the antibiotic was introduced prior to treatment^25–27^. Similar outcomes were observed when type I and type II CRISPR–Cas systems were engineered to target temperate phages that produce antibiotic-resistant lysogens^28,29^. In this manner, indiscriminate targeting of beneficial elements might impede the distribution of CRISPR–Cas systems in natural populations.

Fitness costs associated with the upkeep of CRISPR–Cas systems, including costs which result from basal expression and the occasional acquisition of toxic spacers that target their host’s chromosome, have also been proposed to influence the distribution of these systems^2,30–32^. In certain cases, toxic chromosomal targeting may result from CRISPR–Cas immunity directed at temperate phages which integrate into the host chromosome as prophage DNA during lysogenic infections^28,29^. We previously demonstrated that the type III-A system from *Staphylococcus epidermidis* RP62a^33^ can avert targeting of integrated lambda-like (lambdoid) prophages and give rise to “conditionally tolerant” lysogens in which the CRISPR–Cas system and prophage target have not been genetically altered^29^. In other words, we observed a stable co-existence between functional type III CRISPR–Cas systems and prophage target DNA in lysogenic hosts. Type III systems are distinct from other DNA-cleaving CRISPR–Cas systems where targeting occurs only at sequences which are transcribed, and requires crRNAs which are complementary to the nascent transcripts^29,34^. The type III-A system of *S. epidermidis* RP62a was further shown to encode a targeting complex with nucleases that license cleavage of RNA in addition to DNA^13^, as well as an auxiliary RNase (Csm6) that can assist in the degradation of phage transcripts during lytic infection^35^. Collectively, these type III-A nucleases allow the system to prevent temperate phage propagation when its targets are transcribed during lytic infection or prophage induction, and yet tolerate prophages while their targets are sufficiently repressed in the chromosome. However, type-III A spacers displaying a perfect match with an endogenous prophage are yet to be found in the completed staphylococcal genomes. Thus, it remains to be determined whether conditional tolerance can influence the long-term persistence of type III-A systems in natural populations of lysogenic Staphylococci, including *S. aureus* lineages that are polylysogenized with more than one prophage.

In this work, we use laboratory derivatives of a model clinical isolate, *S. aureus* Newman^36^, and show that maintenance of conditionally tolerant type III-A systems can become a fitness liability for lysogenic hosts. We also provide evidence that the costs result from transcription-dependent targeting at prophage loci, and investigate the effects of different spacers within populations of single or double lysogens.

## Results

### Incomplete prophage tolerance by type III-A systems

**Fig. 1:**
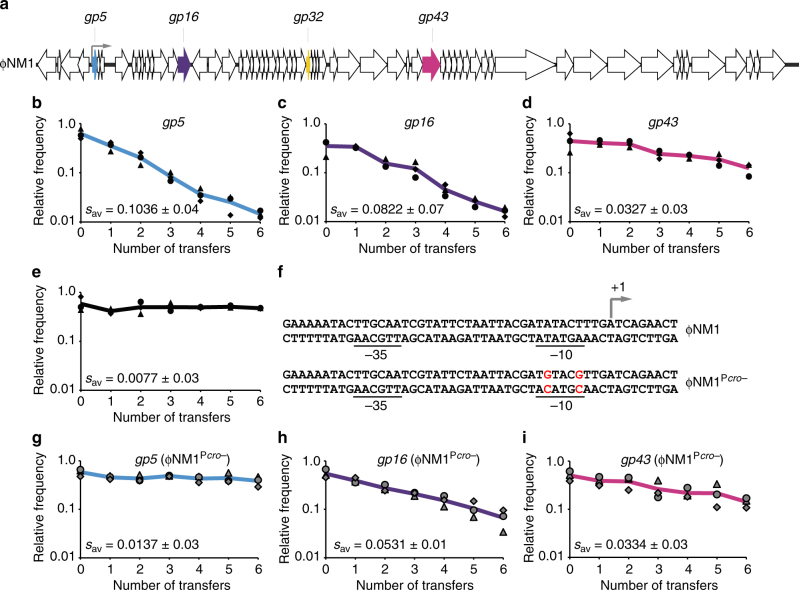
Incomplete prophage tolerance by type III-A systems can be licensed by prophage-internal promoter activity. **a** Schematic representation of the integrated ΦNM1 prophage genome. The *gp5*, *gp16*, *gp32*, and *gp43* open reading frames are colored to denote inclusion of a target sequence for one or more spacers tested in this work. Gray bent arrow indicates the position of ΦNM1’s early lytic promoter (P_*cro*_). **b**–**d** Pairwise competition experiments for three perfectly matching spacers that license conditional tolerance of ΦNM1. In each case, an Erm^R^-marked control lysogen harboring the parent vector with non-targeting spacers was competed against a Tet^R^-marked conditionally tolerant lysogen. Relative frequencies (*y*-axis) are plotted against the number of transfers (*x*-axis), with one transfer per day. Individual values from each biological replicate (*n* = 3) are depicted in black as a triangle, circle, or rhombus. Solid lines represent the average change in relative frequency across the three replicates, and share color coding with the target genes outlined in **a**. **e** Control co-culture experiment. Pairwise competition was performed as in **b**–**d** above, except that differentially marked TB4::ΦNM1 lysogens both harbor the same parent vector with non-targeting spacers. **f** Sequences of the wild-type ΦNM1 early lytic promoter and its inactive ΦNM1^P*cro−*^ variant with point mutations (red lettering) in the −10 element. The location of this promoter, along with the approximate position of its transcriptional start site (+1), was inferred from RNA-sequencing analysis of ΦNM1 during lytic infections^29^. This designation is further supported by research on *S. aureus* lambdoid phages with related regulatory architecture^77–79^. **g**–**i** Pairwise competition experiment as in **b**–**d**, except that targeting and control lysogens both contain the ΦNM1^P*cro−*^ prophage mutations. All *s*_av_ values are derived from Supplementary Table 1 and provide mean ± standard deviation (s.d.)

In a previous work^29^, the CRISPR–Cas system of *S. epidermidis* RP62a was re-engineered to contain spacers targeting the temperate phages of *S. aureus* Newman, and introduced into heterologous RN4220^37^ or TB4 hosts^38^ on pC194-based plasmids^39^. TB4 is a prophage-cured derivative of Newman that can be re-infected with the native phages, including ΦNM1 and ΦNM4. One of our previously tested spacer sequences was modeled from a spacer occurring naturally in the type III-A system of *S. argenteus* MSHR1132^40^, and was found to license conditional tolerance regardless of its five mismatches (Supplementary Fig. 1a) relative to ΦNM1 phage DNA target sequences^29^. When a type III-A plasmid programmed with a perfectly matching variant of this spacer (gp32*) was introduced into TB4::ΦNM1 lysogens, a small colony phenotype was clearly apparent when compared with isogenic lysogens containing the *gp32* or non-targeting CRISPR–Cas plasmids (Supplementary Fig. 1b). We hypothesized that this phenotype reflects a general growth rate reduction relative to non-targeting lysogens, and sought to quantify it more precisely in terms of a spacer-dependent fitness cost. This was accomplished by marking the TB4::ΦNM1 lysogens with small plasmids^41,42^ conferring resistance to either tetracycline (Tet) or erythromycin (Erm), and measuring their relative abundances over time in pair-wise competition co-cultures. As anticipated from the differences in colony size, lysogens with the perfectly matching gp32* spacer seemed to decline in relative abundance more rapidly than lysogens with the mismatched spacer, when each were competed against the non-targeting lysogen (Supplementary Fig. 1c, d). Supplementary Table 1 lists average selection coefficients (*s*, where *s*<0 and *s>*0 imply that the targeting lysogens are at a fitness advantage or fitness disadvantage, respectively), calculated for each competition experiment performed in this study. We measured higher selection coefficients with the perfectly matching gp32*, but this difference was not statistically significant (Supplementary Table 2; linear mixed effect model (lme), *p* = 0.0983). A summary of the statistical tests employed in this study is provided in Supplementary Table 2. To probe the generality of spacer-dependent fitness costs, we tested three additional spacers with perfect identity to sequences within *gp5*, *gp16*, and *gp43* of ΦNM1 (Fig. 1a), and assayed them for relative fitness using the same setup. Significant fitness costs were measured for each of the conditionally tolerant lysogens when their selection coefficients were compared with those of control co-cultures (Fig. 1b–d; lme, *p* < 0.05), in which both competing lysogens harbored the same non-targeting spacers and no significant changes in relative abundance were observed over time (Fig. 1e; one-way ANOVA, *p* = 0.6342).

Previous studies have demonstrated that chromosomal targeting by CRISPR–Cas systems in bacteria is detrimental to growth and potentially lethal^27,30^, even in cases where prophage loci are targeted^28,29,43^. Type III-A systems can tolerate intact chromosomal targets, but only while they are sufficiently repressed. In light of these findings, we hypothesized that the spacer-dependent fitness costs could be derived from low levels of type III-A chromosomal targeting, licensed by transcription at prophage loci. Transcription of prophage loci can occur in a subset of cells during spontaneous prophage induction^44^. However, spontaneous prophage induction is lethal in the absence of targeting spacers, and it is therefore unexpected that non-targeting lysogens would be more fit than lysogens with targeting spacers, as we observed in our competition experiments (Fig. 1). Furthermore, we determined that all of these targeting spacers can disrupt phage lytic propagation via spontaneous (Supplementary Fig. 2a) or MMC-stimulated (Supplementary Fig. 2b) prophage induction. Therefore, if the fitness costs were derived from targeting effects which occur during prophage induction, each spacer might be expected to produce similar fitness costs. Instead, we observed significantly different fitness costs for at least one of the spacers we tested across six transfers (Fig. 1b–d; one-way ANOVA, *p* = 0.0499). To rule out the possibility that prophage induction is required for the fitness defects observed in competition assays, we disrupted ΦNM1’s inducibility by introducing a serine to alanine (S124A) missense mutation in the catalytic domain of its *cI*-like repressor, generating ΦNM1^*ind*−^ (Supplementary Fig. 2c). The CI-like repressors of lambdoid phages possess a serine protease activity that is required for prophage induction via RecA*-stimulated autoproteolysis and de-repression^45–47^. This mutation disrupted both spontaneous (Supplementary Fig. 2a) and MMC-triggered (Supplementary Fig. 2d) prophage induction. Although we were unable to isolate stable transductants of TB4::ΦNM1^*ind−*^ harboring the *gp5*-targeting plasmid, fitness costs associated with each of the *gp16*- and *gp43*-targeting plasmids were not significantly reduced in this prophage-mutant background (Supplementary Fig. 2e–,f; lme, *p* = 0.9786 and *p* = 0.5431, respectively), when selection coefficients were compared to those obtained in the wild-type background (Fig. 1c, d).

The results above confirm that de-repression during prophage induction is not required for fitness costs associated with conditionally tolerant type III-A systems in lysogens. We therefore postulated that the costs could be licensed by unregulated transcription from one or more promoter within the target prophage. To explore this possibility, we introduced inactivating mutations into the rightward promoter of ΦNM1 (Fig. 1a, f) that is predicted to be involved in the lysis/lysogeny decision of lambdoid phages^48^, generating ΦNM1^P*cro*−^. As expected, mutation of this promoter was sufficient to abolish the prophage’s lytic cycle in two assays (Supplementary Figs. 2a and g), so we proceeded to test these mutant lysogens in competition co-cultures. The P_*cro*_ mutation virtually abolished the fitness cost associated with the *gp5* spacer, which targets a sequence immediately downstream of the promoter, such that selection coefficients were no longer significantly higher than those obtained in control competitions (Fig. 1g; lme, *p* = 0.2721). In contrast, the costs associated with the *gp16* and *gp43* spacers were only partially reduced or not significantly reduced at all, respectively, when compared to their costs in the wild-type ΦNM1 background (Fig. 1h, i; lme, *p* = 0.0321 and *p* = 0.6711, respectively). Potentially, these fitness costs are licensed by leaky transcription from other sequences in the prophage genome. Taken together, these results demonstrate that conditionally tolerant type III-A systems can impose spacer-dependent fitness costs even if their target prophages are not inducible, and rather suggest that the costs arise from leaky transcription of prophage targets within otherwise stably lysogenic cells.

### Contributions of targeting nucleases to fitness costs

**Fig. 2:**
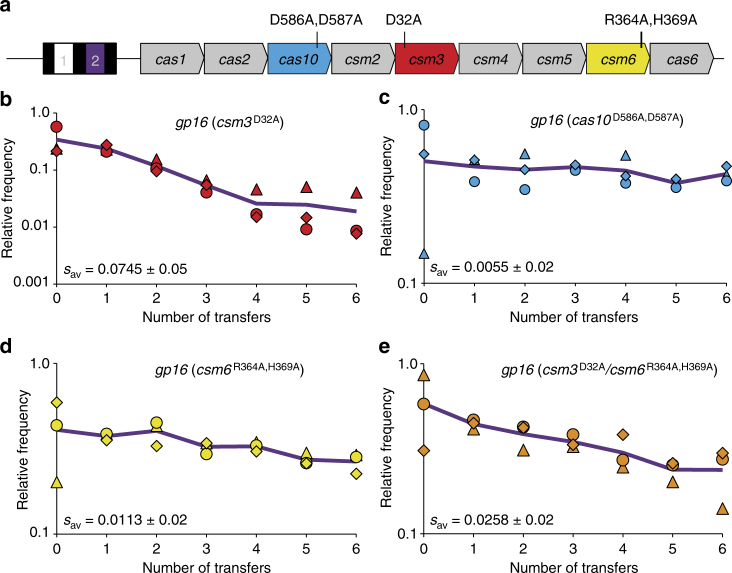
Effect of targeting nuclease active site mutations on fitness costs associated with type III-A systems. **a** Schematic diagram summarizing the arrangement of CRISPR–Cas loci used throughout the work, with position 2 of the CRISPR array occupied by the *gp16* spacer (purple rectangle) in this example. The naturally occurring *nes* spacer (white rectangle) from *S. epidermidis* RP62a is maintained at position 1 in all cases. Open reading frames (ORFs) encoding Cas nucleases implicated in targeting are highlighted in blue (*cas10*), red (*csm3*), or yellow (*csm6*), and the positions of their active site mutations are labeled to scale within each ORF. Other features of the diagram are not drawn to scale. **b**–**e** Pairwise competition experiments for mutant CRISPR–Cas plasmids harboring the *gp16* spacer with perfect matches to ΦNM1. In each case, an Erm^R^-marked control lysogen harboring the wild-type parent vector with non-targeting spacers was competed against a Tet^R^-marked lysogen harboring a mutant CRISPR–Cas plasmid with the *gp16* spacer. Relative frequencies (*y*-axis) are plotted against the number of transfers (*x*-axis), with one transfer per day. Individual values from each biological replicate (*n* = 3) are depicted as a triangle, circle, or rhombus, and are colored to match the ORFs highlighted in **a** for each mutant, or orange for the *csm3*/*csm6* double mutant. Solid lines represent the average change in relative frequency across the three replicates, and are colored purple to match the *gp16* spacer’s target gene as outlined in Fig. 1. The *s*_av_ values are derived from Supplementary Table 1 and provide mean ± s.d

Having demonstrated that lysogenic hosts which maintain type III-A systems can incur spacer-dependent fitness costs, we wanted to determine whether this phenomenon results from CRISPR–Cas targeting. To this end, we measured fitness costs associated with the *gp16* spacer in the presence of mutations in Cas nuclease active sites previously implicated in targeting by the *S. epidermidis* type III-A system (Fig. 2a). Mutation of Csm3 (D32A) was previously shown to abolish crRNA-guided transcript cleavage, while mutation of Cas10’s palm polymerase domain (D586A, D587A) was shown to abolish the complex’s capacity for transcription-dependent cleavage of DNA^13^. In addition, mutations in the HEPN domain of the Csm6 RNase (R364A, H369A) were shown to abolish its transcript degradation activity and eliminate its contributions to phage defense^35^. Whereas no significant fitness cost reduction was detected with the *csm3* mutant when compared to the wild-type plasmid harboring *gp16* (Fig. 2b; lme, *p* = 0.5655), fitness costs appeared to be completely abolished with the *cas10* palm domain mutant (Fig. 2c), given that no significant difference was observed when selection coefficients were compared with that of control competitions (lme, *p* = 0.5341). Notably, both the *csm6* and *csm3*/*csm6* mutants also displayed a significantly reduced fitness cost, compared to the wild-type *gp16* plasmid (Fig. 2d, e; lme, *p* = 0.0026 and *p* = 0.0272, respectively). These findings imply that the targeting activities of Cas10 and Csm6 contribute to growth defects associated with incomplete tolerance of prophages by type III-A CRISPR–Cas systems.

### Consequences of conditional targeting in polylysogens

**Fig. 3:**
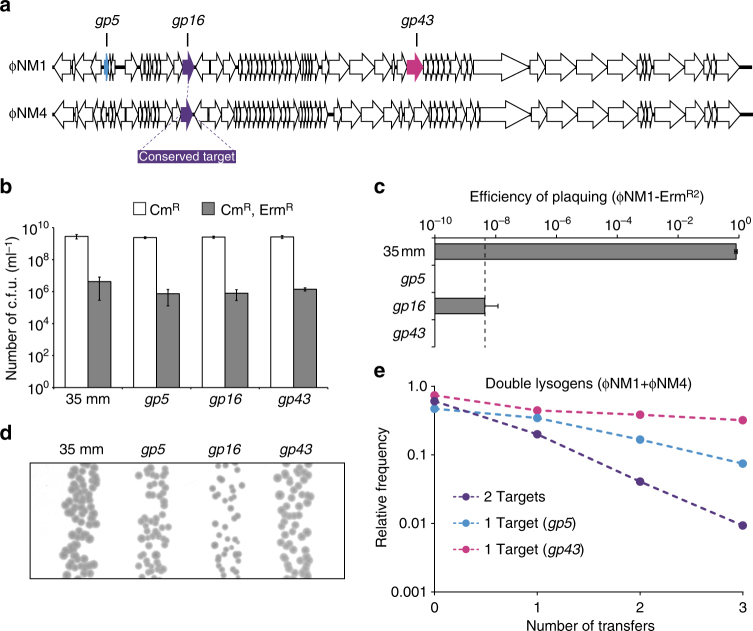
Type III-A systems which target multiple temperate phages can impose greater fitness costs in polylysogenic hosts. **a** Schematic representation of the integrated ΦNM1 and ΦNM4 prophage genomes. Colored open reading frames (ORFs) denote the presence of a target sequence for perfectly matching spacers. ΦNM1’s *gp16* sequence includes a target that is found with 100% conservation in *gp17* of ΦNM4, as indicated by color-matched ORFs (purple). **b** Secondary lysogenization of TB4::ΦNM4 with ΦNM1-Erm^R2^ in the presence of the 35 mm, *gp5*, *gp16*, or *gp43* CRISPR–Cas plasmids as indicated. The “35 mm” spacer is a fully mismatched variant of the *gp16* spacer that serves as a non-targeting control. Lysogenization was quantified as the concentration of Cm^R^+Erm^R^ colony-forming units (colony-forming units per milliliter) in each culture following treatment with ΦNM1-Erm^R2^. For comparison, total recipients were quantified as the concentration of Cm^R^ colony-forming units in each untreated culture. Error bars, mean ± s.d. (*n = *3, biological replicates). **c** Plaquing efficiency of ΦNM1-Erm^R2^ on TB4::ΦNM4 lysogens harboring the different CRISPR–Cas plasmids tested in **b**, as indicated by the labels. Efficiency ratios were calculated for each plasmid relative to plaques quantified on a TB4 lawn harboring the non-targeting parent vector. Dotted line represents the limit of detection under these assay conditions. Error bars, mean ± s.d. (*n = *3, technical replicates). **d** Colony size comparison of TB4::ΦNM4+ΦNM1-Erm^R2^ double lysogens harboring the different CRISPR–Cas plasmids tested in **b**, as indicated by the labels above. Picture is representative of a biological replicate for each strain plated in the presence of chloramphenicol to maintain CRISPR–Cas plasmids. **e** Batch competition experiment with unmarked TB4::ΦNM4+ΦNM1 double lysogens harboring the different CRISPR–Cas plasmids tested in **b**. As outlined in the methods, deep sequencing was used to determine targeting spacer frequencies relative to the 35 mm non-targeting spacer within the sample (*y*-axis), and plotted against the number of transfers (*x*-axis, one transfer per day). Dashed lines illustrate the change in relative frequency across time points, and are color-coded for their targets according to the schematic in **a**

Clinical isolates of *S. aureus* are seldom found to possess CRISPR–Cas systems^49^, and in many cases are polylysogens harboring more than one prophage^50,51^. The clinical isolate *S. aureus* Newman, for example, does not harbor an endogenous CRISPR–Cas system but harbors four heteroimmune lambdoid prophages^38^. However, among the staphylococcal type III-A CRISPR–Cas systems identified so far some spacers have been found to match conserved temperate phage lytic genes^29,49^, and presumably offer the potential to target multiple phages. These observations prompted us to investigate the consequences of maintaining type III-A systems in a polylysogenic scenario where more than one prophage can be targeted. This was accomplished using ΦNM1 and ΦNM4, which integrate into distinct chromosomal loci but share extensive regions of sequence homology, including a perfectly conserved target for the *gp16* spacer (Fig. 3a). Provided that each target sequence falls within lytic gene clusters which are sufficiently repressed during lysogeny, we anticipated that polylysogeny would be tolerated even in the presence of perfectly matching spacers. To confirm this, TB4::ΦNM4 lysogens harboring CRISPR–Cas plasmids with spacers matching to ΦNM1’s *gp5*, *gp16*, or *gp43* targets were infected with an erythromycin resistance-conferring derivative of ΦNM1 (ΦNM1-Erm^R2^) that allows quantification of newly formed lysogens. When compared with control lysogens harboring a fully mismatched (35 mm) spacer, we found no significant differences in the frequency of ΦNM1-Erm^R2^ secondary lysogenization for lysogens with the singly (*gp5*, *gp43*) and doubly (*gp16*) targeting spacers (Fig. 3b; one-way ANOVA, *p* = 0.1906), while plaque formation on the same hosts was strongly reduced in the presence of targeting spacers (Fig. 3c). However, differences in colony size phenotype were clearly discernible among the resulting double lysogens. Relative to double lysogens with the control spacer, double lysogens with the *gp5* or *gp43* spacers which do not target ΦNM4 had a less severe colony size reduction than lysogens with the *gp16* spacer targeting both prophages (Fig. 3d). To determine whether these phenotypes reflect discrepancies in relative fitness, we mixed unmarked double lysogens harboring each of the three spacers into a single batch and competed them in liquid culture for 3 days. At each transfer interval, spacer abundances were measured by deep sequencing, and the frequency of each targeting spacer was calculated relative to the control spacer’s frequency in the sample. These pairwise relative frequencies were then used to calculate selection coefficients for each strain (Supplementary Table 1). Our analysis indicates that the fitness cost associated with the dual-targeting *gp16* spacer in double lysogens was significantly greater than that obtained with the *gp5* and *gp43* spacers targeting only ΦNM1 (Fig. 3e; one-sided *t* tests, *p* = 0.0095 and *p* = 0.0049, respectively). Importantly, although the *gp16*-associated cost in double lysogens was significantly higher than its average cost in pairwise competitions with either of the marked TB4::ΦNM1 (Fig. 1c; one-sided *t* test, *p* = 0.0056) or TB4::ΦNM4 single lysogens (Supplementary Fig. 3; one-sided *t* test, *p* = 0.0095) across the first three transfers, we did not observe a significant difference when comparing the *gp5*- or *gp43*-associated costs in double lysogens (Fig. 3e) to their average costs in pairwise competitions with marked TB4::ΦNM1 single lysogens across the first three transfers (Fig. 1b, d; two-sided *t* tests, *p* = 0.6322 and *p* = 0.5643). These results demonstrate that type III-A CRISPR–Cas systems can become a greater fitness liability if they target multiple temperate phages, despite their capacity for conditional tolerance.

### Effects of spacer-target mismatches on fitness costs

**Fig. 4:**
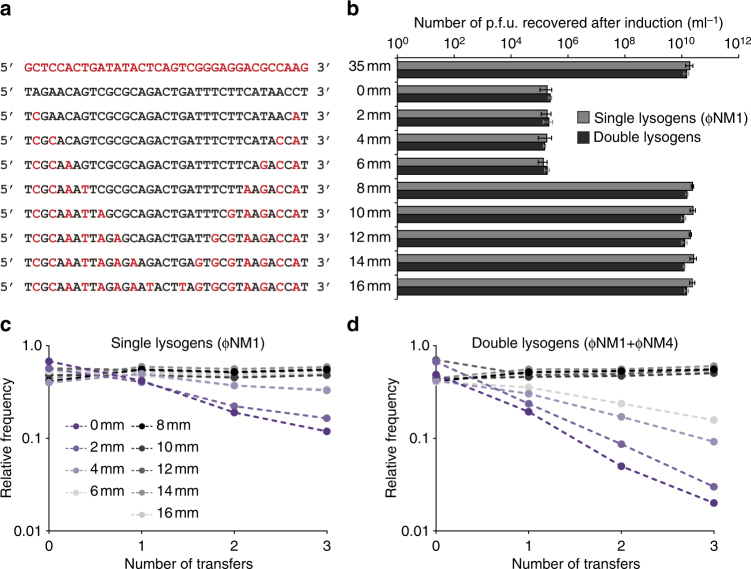
Effects of spacer-target mismatches in single and double lysogens harboring a CRISPR–Cas system with dual-targeting potential. **a** Sequence summary of the *gp16* spacer and its mismatched variants. Black lettering indicates sequences found in the parental *gp16* spacer; red lettering indicates mutated base pairs. A subset of transversion mutations were used to avoid introducing G:U pairing when complementarity between the crRNA and transcribed target RNA is considered. **b** Quantification of plaque forming unit concentrations (plaque-forming units per milliliter) in filtered supernatants from subcultures of single (TB4::ΦNM1) or double (TB4::ΦNM4+ΦNM1) lysogens harboring the spacers depicted in **a**, recovered 4 h post treatment with Mitomycin C at 1.0 µg ml^−1^. Error bars, mean ± s.d. (*n = *3, biological replicates). **c** Batch competition experiment with unmarked TB4::ΦNM1 single lysogens harboring CRISPR–Cas plasmids with the perfectly matched *gp16* spacer or one of several mismatched derivatives shown in **a**. As outlined in the methods, deep sequencing was used to determine targeting spacer frequencies relative to the 35 mm spacer within the sample (*y*-axis), and plotted against the number of transfers (*x*-axis, one transfer per day). Dashed lines illustrate the change in relative frequency across time points, and are color-coded according to the labeled inset. **d** Same as in **c**, except that unmarked TB4::ΦNM4+ΦNM1 double lysogens were used in the experiment

The above results confirm that type III-A systems that target multiple temperate phages are not a barrier to polylysogenization per se, even if they possess perfectly matching spacers. However, when searching the fully sequenced staphylococcal genomes which were previously found to contain type III-A systems^49^, we were unable to identify cases where perfectly matching targets co-existed with spacers within the same genome. Meanwhile, as previously reported, one of these genomes possesses a spacer with two partially mismatched targets (each within a separate prophage), and we were able to confirm this using modified search parameters. Having found, also, that our partially mismatched *gp32* spacer is still capable of targeting ΦNM1 during the lytic cycle (Supplementary Fig. 2) but displayed a fitness cost which appeared to be lower (although this reduction was not statistically significant) than that of its perfectly matched gp32* variant in single lysogens (Supplementary Fig. 1), we wondered if spacer-target mismatches could likewise help to minimize fitness tradeoffs in polylysogenic populations. To investigate this, we constructed a series of mismatched spacers based around our *gp16* spacer, which targets an identical sequence found in both ΦNM1 and ΦNM4 (Fig. 3a). It was previously shown that mismatches most readily abrogate type III targeting when they are distributed at both the 5′ and 3′ ends of the spacer^49,52^. Accordingly, we introduced increasing numbers of mutations at both ends of the *gp16* spacer (Fig. 4a) in an effort to find a minimal number of mismatches that reduces fitness costs without eliminating targeting during the lytic cycle. The resulting CRISPR–Cas plasmids were then transduced into unmarked TB4::ΦNM4+ΦNM1 double lysogens, as well as unmarked TB4::ΦNM1 single lysogens for comparison. We first examined the potential of each spacer to disrupt the phage lytic cycle in single and double lysogens during MMC-stimulated prophage induction. In both cases, the number of plaque-forming units recovered after induction was reduced to a similar degree by the spacers with six or fewer mismatches, while targeting was undetectable with eight or more mismatches (Fig. 4b). The *gp16* variant with six mismatches was also confirmed to be protective in lytic infection assays with ɸNM1γ6^29^, a virulent mutant of ɸNM1 (Supplementary Fig. 4a, b). In particular, when comparing the average fold change in relative abundance before and after infection of competition co-cultures (Supplementary Fig. 4c), we observed no significant difference for the isogenic strains with zero- and six-mismatched spacers (0.5982 ± 0.09 and 0.7842 ± 0.02, respectively; two-sided *t* test, *p* = 0.0669). Evidently, survival was not impaired by the presence of even six spacer-target mismatches during the course of the experiment. These results are well in line with previous reports demonstrating that our type III-A system’s in vivo targeting requirements depend on such factors as mismatch position, sequence context, and assay conditions; in some cases even ten spacer-target mismatches can be introduced before targeting of transcribed DNA elements is abrogated, while in other cases as few as three are sufficient to abrogate targeting^29,35,53,54^.

We next tested each group of single or double lysogens in batch competition experiments. Again, spacer abundances were measured by deep sequencing at each transfer interval for 3 days, and the frequency of each spacer was calculated relative to the fully mismatched control spacer’s frequency in the sample. Our analysis indicates that the fitness costs were significantly reduced for spacers with four or six mismatches when compared to those of the perfectly matching *gp16* spacer within their respective populations of single (Fig. 4c; one-sided *t* tests, *p* = 0.0280 and *p* = 0.0290, respectively) or double (Fig. 4d; one-sided *t* tests, *p* = 0.0436 and *p* = 0.0281, respectively) lysogens. As expected from the observed lack of targeting during the phage’s lytic cycle, no significant costs were measured for any of the spacers with eight or more mismatches in either population (one-way ANOVAs, *p* = 0.7408 and *p* = 0.6265, respectively), when selection coefficients were compared with that of the average obtained in control TB4::ΦNM1 pairwise competitions across their first three transfers (Fig. 1e). Importantly, we observed no significant difference between the cost associated with the perfectly matching *gp16* spacer in competitions with marked and unmarked TB4::ΦNM1 single lysogens (Fig. 1c and 4c; two-sided *t* test, *p* = 0.3862), and this served to validate our usage of deep sequencing to assess relative fitness. We also found that the cost associated with the perfectly matching *gp5* spacer targeting only ΦNM1 (Fig. 3e) was not significantly different from the costs measured for the dual-targeting spacers in double lysogens possessing either four or six mismatches (Fig. 4d; two-sided *t* tests, *p* = 0.4508 and *p* = 0.1433, respectively). Altogether, these results confirm that spacer-target mismatches can alleviate fitness costs associated with incomplete prophage tolerance by type III-A systems in (poly)lysogenic staphylococci, even in cases where immunity to lytic infections is not abrogated.

## Discussion

We report here that certain conditionally tolerant type III-A CRISPR–Cas systems can put their (poly)lysogenic hosts at a fitness disadvantage in mixed populations of *S. aureus*, despite the potential for these CRISPR–Cas systems and their prophage target(s) to co-exist in (poly)lysogenized clones. The fitness costs varied in severity with the position of targets within the prophage genome, and likely result from incomplete prophage tolerance by the system’s transcription-dependent targeting machinery. We previously showed that transcription-dependent chromosomal targeting by the type III-A system of *S. epidermidis* RP62a causes severe growth defects^29,35^, and similar effects have been observed with other types of CRISPR–Cas systems^27,28,30^. At least in part, this appears to result from toxicity associated with frequent nicking or cleavage of the host’s chromosomal DNA. Minor growth defects, in line with the fitness costs described in this work, might instead be expected if type III-A chromosomal targeting were infrequent; e.g., licensed by transcription in a small subpopulation of cells. When lambdoid lysogens are cultured under standard growth conditions, transcription from lytic promoters can occur in a subpopulation of cells undergoing the lytic cycle via spontaneous prophage induction^44^. However, our results suggest that the fitness costs might depend instead on leaky transcription of prophage targets occurring within stably lysogenic cells, rather than transcription occurring in a subpopulation of cells that is undergoing spontaneous prophage induction (Supplementary Fig. 2). It is also important to note that due to the use of a high copy plasmid-based type III-A CRISPR–Cas system, the fitness costs we observed may be exacerbated due to a higher expression of this system. Consistent with a requirement for transcription at prophage target loci, costs associated with the *gp5* spacer were virtually undetectable when the P_*cro*_ promoter immediately upstream of its target was inactivated (Fig. 1g). In contrast, mutation of P_*cro*_ did not eliminate fitness costs associated with spacers that target the *gp16* and *gp43* prophage loci (Fig. 1h, i). This latter finding suggests that the fitness costs associated with these spacers are caused by leaky transcription from other promoter sequences within the prophage genome. The *gp43* target is located within a gene cluster that is transcribed late during the lytic cycle of ΦNM1^29^. In staphylococcal phages with related genomic architecture, this cluster lies downstream of a regulatory region that was shown to contain a conserved transcriptional terminator^55^, and we identified such a region in ΦNM1 between *gp36* and *gp37*. Conceivably, the magnitude of the *gp43* spacer’s fitness cost is determined by one or more late promoters which are insulated from upstream transcription during the non-inducing conditions of lysogeny, perhaps in part due to the terminator in this regulatory region.

The potential for prophage lytic regions to be de-repressed independently of spontaneous prophage induction is not unprecedented for lambdoid temperate phages, and was previously demonstrated using a genetic reporter system^46^. In this previous study, the authors detected slight de-repression above background even when prophages carried a non-inducible *cI* allele similar to that of our ΦNM1^*ind−*^ mutant. Leaky transcription arising in this manner could likewise be responsible for the fitness costs detected in our study. A model such as this would perhaps explain the exacerbated fitness cost observed with double lysogens where both prophages were targeted with the *gp16* spacer (Fig. 3e), if we assume that transcription of each prophage is only well-coordinated in cells undergoing prophage induction^56^. In this scenario, if instances of leaky transcription in each prophage were poorly correlated within individual cells, the combined effect of having both targets might essentially be additive at the population level. Consistent with this idea, the cost associated with the *gp16* spacer was found to be greater in double lysogens than in single lysogens of ΦNM1 and ΦNM4 alone (Fig. 1c and Supplementary Fig. 3a). Examples of uncorrelated leaky expression from distinct chromosomal loci in bacteria are also not unprecedented. It was previously demonstrated that cell–cell variability becomes elevated under transcriptionally repressed conditions, even when expression from two loci with identical promoters and identical repressors is compared^57^. Similar single-cell approaches could be used to clarify whether expression of the two prophages used in this study is occasionally uncoupled.

Additional evidence supporting transcription-dependent chromosomal targeting as the source of the fitness costs was provided by experiments with mutant type III-A Cas nucleases. Ablation of *csm3*’s RNase activity did not reduce fitness costs (Fig. 2b). Given its role in specific cleavage of target RNAs by type III systems^13,58,59^, this was perhaps expected; knockdown of prophage lytic transcripts should not be toxic to lysogenic cells per se. Meanwhile, fitness costs appeared to be completely abolished by mutation of *cas10*’s palm polymerase domain (Fig. 2c), in line with past reports indicating that transcription-dependent DNA degradation by this system requires an intact palm polymerase domain^13^. Interestingly, mutation of *csm6*’s HEPN RNase domain strongly reduced the cost associated with the *gp16* spacer as well (Fig. 2d, e). Our previous study^35^ showed that Csm6’s RNase activity contributes to the degradation of phage lytic transcripts, and this should not necessarily be toxic for lysogens. However, given that the specificity of Csm6’s RNA knockdown effect has not been fully determined, it is possible that this activity could cause occasional off-target cleavage of essential host transcripts, and detectably impact fitness in our competition assays. Of relevance here, it was recently shown that the related HEPN domains of type VI effector RNases can mediate cleavage of non-target transcripts, or “collateral” RNA cleavage, in the presence of target RNAs^8^. Growth defects associated with type VI targeting in vivo were accordingly proposed to result from toxic off-target cleavage of cellular transcripts^60,61^. More recently, binding of target RNAs by type III complexes was found to enable synthesis of a cyclic oligonucleotide that stimulates the RNase activity of Csm6 proteins, and this depended on the Cas10 subunit’s intact palm polymerase domain^62,63^. The phenotypic similarity observed with our *cas10* and *csm6* mutants in fitness assays (Fig. 2) might therefore be explained, at least in part, by a dominant effect of the *cas10* palm domain mutation.

The results presented in this work suggest that, among (poly)lysogenization-prone populations of *S. aureus* ^50,51^, natural selection could favor the persistence of conditionally tolerant type III-A systems with spacers that are partially mismatched relative to their temperate phage targets. In line with this possibility, a putative conditionally tolerant isolate of *S. aureus* was recently reported to contain five mismatches in each prophage target relative to a spacer in its type III-A CRISPR–Cas system^49^. Conceivably there are various evolutionary routes that could lead to such co-existences between a type III-A system and one or more partially mismatched prophage targets. Although the spacers acquired upon phage infection during the adaptive stage of the CRISPR–Cas immune response are expected to match their original targets with perfect complementarity^3^, conditionally tolerant type III-A spacers can allow for stable lysogenization even if they are perfectly matching^29^. However, as our current work illustrates, the resulting conditionally tolerant (poly)lysogens can suffer fitness costs. In principle, mutations which alleviate these fitness costs could progressively accumulate in the spacer or prophage target sequence(s) after (poly)lysogeny is established. This, however, is unlikely to happen given the low frequency of spontaneous mutation in bacteria^64,65^. In addition, most of the cells that acquire a new spacer during phage infection will not become lysogens (depending on the phage and host, the lysogenization frequency in staphylococci varies between 10^−3^ and 10^−4^). We believe that these non-lysogens could provide an alternative route for the establishment of conditionally tolerant mismatched spacers with minimal fitness costs. Due to the tolerance of spacer-target mismatches that is characteristic of type III-A CRISPR–Cas immunity^29,35,53,54^, the new spacer could provide efficient defense against related temperate phages with incomplete homology with their target sequence. If infection with such phage were to result in type III-A conditional tolerance and lysogenization, the spacer will contain mismatches with the prophage target that could lead to its persistence without a fitness cost. Interestingly, in *Pseudomonas aeruginosa*, it was previously found that five spacer-target mismatches could enable co-existence between a type I-F system and its prophage target, although these mismatches abolished immunity to lytic infections^43^. Intriguingly, chromosomal targeting effects were not completely abrogated in that scenario, and were sufficient to drive a host-encoded response that inhibits group behaviors in surface-grown populations^66,67^. Whether or not type III-A chromosomal targeting could give rise to a similar environment-specific phenotype remains to be investigated. Finally, it is important to note that type III-A systems have also been found to display signatures of horizontal acquisition within *S. aureus* genomes^49,68,69^. Hence, we further speculate that the genetic stability afforded by conditional tolerance in the presence of spacer-target mismatches, could be achieved through the horizontal dissemination of prophage-targeting type III-A systems into (poly)lysogenic populations harboring prophages containing mismatches with the targeting spacers. Additional investigation will be required to establish how these features of type III-A temperate phage targeting influence fitness in actively CRISPR-adapting populations, where spacer diversity is potentially much greater.

## Methods

### Bacterial strains and growth conditions

Unless otherwise noted, cultivation of *S. aureus* RN4220^37^ or TB4^38^ and their derivatives was carried out in sterile 15 or 50 ml conical-bottom Falcon tubes (Corning) containing Difco TSB liquid media (volume ≤ 14% tube capacity), or on sterile petri dish plates containing Difco TSA solid media. Media was supplemented with erythromycin (Erm) at 10 µg/ml or tetracycline (Tet) at 5 µg/ml only when streaking out single colonies of marked TB4 derivatives containing pE194^41^ or pT181^42^ plasmids, respectively, and during the construction of these strains as needed. All media was supplemented with chloramphenicol (Cm) at 10 µg/ml when cultivating strains with CRISPR–Cas plasmids, and/or CaCl_2_ 5 mM if the media was intended to support phage infection and transduction. Unless otherwise noted, plates were incubated at 37 °C for 12–18 h and then stored at 4 °C for up to a week. Overnight liquid cultures were inoculated from single colonies (biological replicates), grown for 12–16 h at 37 °C with shaking, and then stored at 4 °C for 3 h or less before briefly vortexing and subculturing at a 1:100 dilution as needed. Where applicable, subcultures were treated with Mitomycin C (AG Scientific) to a final concentration of either 1.0 or 2.0 µg/ml, or the virulent phage ɸNM1γ6 at a multiplicity of infection ~7.5, as indicated in the figure legends. When the overnight cultures were to be used in multi-day competition experiments, 24 h of growth was instead allowed, followed by immediate vortexing, mixing of aliquots, and passaging at a 1:1000 dilution. When streaking out lysogens with targeting spacers, aberrantly large colonies were presumed to have lost conditional tolerance either via genetic inactivation of CRISPR–Cas plasmids or prophage deletion, and not studied further in this work.

### DNA preparation and cloning

Plasmid DNA of *E. coli* DH5α was purified from 4 to 6 ml overnight cultures using plasmid miniprep reagents from Qiagen, according to the manufacturer’s protocol. DNA from *S. aureus* RN4220 or TB4 was purified similarly except that 2 ml overnight cultures were used, and cells were treated with 10–15 µl lysostaphin (1 mg ml^−1^) at 37 °C for 1.5 h immediately after resuspension in P1 buffer. Minipreps were carried out with either Qiagen or EconoSpin columns. For PCR-based cloning procedures, DNA was amplified with Phusion polymerase (Thermo) and purified using Qiagen reagents and EconoSpin columns. When generating amplicons for Sanger sequencing, TopTaq polymerase (Qiagen) was often used for amplicons smaller than 750 bp, and DNA was not necessarily purified after the PCRs.

Electrocompetent RN4220 cells (described previously^29^) were used for cloning in *S. aureus*. The pGG79 parent vector (Supplementary Table 3) is a derivative of pGG3-BsaI^29^ with the BsaI placeholder sequence at position two trimmed at its 3’ end by one base pair, and a CRISPR repeat inserted immediately downstream. This was accomplished by inverse PCR^70^ using primers oGG281/oGG282 (Supplementary Table 4), followed by blunt ligation. The CRISPR array was sequenced by Sanger as described previously^29^, and the expected plasmid size was verified via analytical digestion during subsequent manipulations. The immune functionality of this plasmid in *S. aureus* was later also confirmed by resistance to infection with ɸNM4γ4α2, a derivative of ɸNM4γ4^24^ possessing a target for the *nes* spacer in position one of pGG79’s CRISPR array. Except where noted, all other CRISPR–Cas plasmids with wild-type *cas* genes were constructed via scarless insertion of spacers between CRISPR repeats 2 and 3 of the parent vector, pGG79. This was accomplished by restriction digesting the two oppositely oriented BsaI sites and then ligating them with an annealed oligonucleotide pair possessing compatible overhangs and the desired spacer sequence (Supplementary Tables 3 and 4). CRISPR array modifications were then verified again as described previously^29^. CRISPR–Cas plasmids with the *gp16* spacer and one or more *cas* gene mutations were also constructed by the annealed oligo cloning method in most cases, and checked similarly, except that mutant parent vectors were used as backbones for insertion of the spacer. The *csm3* (D32A) mutant vector, pPS95, was constructed via 2-piece Gibson assembly of PCR products^71^ amplified from pGG79 using primers PS153/PS465 and PS154/PS466. The *csm6* (R364A, H369A) mutant vector, pGG99, as well as the *csm3/csm6* double mutant vector, pGG89, were also constructed via 2-piece Gibson. For pGG99, the fragment containing the CRISPR array was amplified from pGG79 using primers W852/PS566, while the rest of the backbone including the *csm6* mutations was amplified from pWJ241^35^ using primers PS565/W614. The same was done for pGG89, except that pWJ242^35^ was used for templating the backbone with both *csm3* and *csm6* mutations. To construct the pGG167 *cas10* (D586A, D587A) palm domain mutant plasmid harboring the *gp16* spacer, 2-piece Gibson was performed with a CRISPR-containing fragment amplified from pGG102 using primers W852/oGG458, and a backbone fragment amplified from pGG139 using primers oGG457/W614. The pGG139 vector contains *cas10* mutations in both the HD (H14A, D15A) and palm polymerase (D586A, D587A) domains, and was itself constructed via 2-piece Gibson of a fragment amplified from pGG79 with W852/oGG425 and a fragment amplified from pAV71 using primers oGG424/W614. Following Gibson assembly of these vectors, the expected plasmid size was confirmed by restriction digest, and regions of interest were sequenced by Sanger using primers listed in Supplementary Table 4 (Gibson assembly junctions, CRISPR arrays, and in select cases the relevant *cas* nuclease active sites). The pAV71 vector was constructed via 2-piece Gibson of a fragment containing the HD domain mutation amplified from pLM546^72^ with W125/PS557, and a fragment containing the palm domain mutation amplified from pWJ291 with PS556/W762. The pWJ291 vector was constructed via 2-piece Gibson of fragments amplified from pWJ30β^29^ using primer pairs W852/W1169 and W1170/W614. JW233 is a derivative of RN4220 with a type III-A CRISPR–Cas plasmid that targets ɸ11’s *cI*-like repressor gene. This plasmid was constructed from pGG3-BsaI according to the annealed oligo cloning procedure described previously^29^, using oligos JW398 and JW399 for insertion of the spacer. The pAV43 plasmid was constructed by 3-piece Gibson assembly of two homology fragments amplified from ɸNM4γ4 (AV205/AV202 and AV203/AV185), along with a backbone fragment amplified from pC194 (AV186/AV204). The pE194-*tyc1* plasmid was constructed by 1-piece Gibson assembly of PCR products generated with primers oGG297/oGG298. The presence of an intact assembly junction, including the 5′ UTR SNP in *ermC*, was subsequently confirmed by Sanger sequencing with primers oGG191/W235. The pGG90 plasmid was constructed by 2-piece Gibson assembly of PCR products; its integrase homology fragment was amplified from ɸNM1 with primers oGG315/oGG316, while its backbone fragment including *ermC* homology was amplified from pE194-*tyc1* using primers oGG314/oGG317. Intact assembly junctions were verified by PCR and Sanger sequencing with primers W234/W235.

Chemically competent DH5α cells were used for cloning pWJ327-derived vectors in *E. coli*, each constructed via 3-piece Gibson assembly of PCR products. The pGG170 plasmid contains homology to ɸNM1 with mutations in the P_*cro*_ promoter, while the pGG172 plasmid contains homology to ɸNM1 with mutations in its *cI*-like repressor. A backbone fragment was amplified from pWJ327 using primers JW809/JW810, while the homology arms were amplified from ɸNM1 using primer pairs oGG467/oGG468 and oGG469/oGG470 for pGG170, or oGG471/oGG472 and oGG473/oGG474 for pGG172. The expected size of the resulting plasmids was confirmed by restriction digest, and homology arm sequences were confirmed by Sanger using primers listed in Supplementary Table 4 (W1250, oGG455, and oGG469 or W1250, oGG471, and oGG473). Unexpectedly, the plasmids failed to transform RN4220. PCR and Sanger sequencing of the region encoding elements required for replication in *S. aureus* at 28 °C revealed a SNP within the temperature-sensitive *repA* gene, resulting in a premature stop codon. The SNP was corrected in each case to create “pGG170ts” or “pGG172ts” via 1-piece Gibson of PCR products amplified from pGG170 or pGG172 using primers oGG509/oGG510, followed by electroporation directly into RN4220 and plating at 28 °C.

### Estimation of phage lysate titers

As needed, phage lysate titers were estimated by spotting serial dilutions on a lawn of sensitive bacteria to enumerate pfu, according to the procedure described previously^29^.

### Preparation of transducing lysates

Overnight culture aliquots (100 µl) of RN4220 or TB4 derivatives harboring a plasmid of interest were infected with “clear” mutant (lytic) derivatives of either ɸNM1, ɸNM2, ɸNM4, or ɸ11 (MOI ~ 0.1–10) in HIA soft agar supplemented with CaCl_2_ at 5 mM, and incubated overnight at 37 °C to produce a lysed lawn. Within 48 h of storage at 4 °C, soft agar was scraped and decanted into a 50 ml conical-bottom Falcon tube, supplemented with an additional 1.2 ml fresh HIB, and then centrifuged at 4303 × *g* for 8–10 min. Filtered supernatants containing the transducing particles were stored in autoclaved 1.5 ml tubes (Eppendorf) at 4 °C. When the plasmid of interest carried a CRISPR–Cas system, care was taken to lyse its host with a suitable non-targeted phage, among the following: ɸNM1γ6^29^, ɸNM2γ1, ɸNM1γ6α1, ɸNM4γ4^24^, or ɸ11γ2β1. Phages not described previously were procured via CRISPR-assisted editing as outlined below.

### CRISPR-assisted editing of phages

Recombinant phages were isolated by using type III CRISPR–Cas plasmids to counter-select against different target sequences. The ɸ11γ2β1 phage is a mutant of ɸ11γ2 isolated during infection of lawns harboring the pGG102 plasmid (*gp16*). After an additional round of counter selection with pGG102, the presence of a 357 bp deletion encompassing the target site was confirmed by PCR and Sanger sequencing with primers oGG387/oGG388. ɸ11γ2 is a clear mutant of ɸ11 that was isolated during infection of JW233 lawns harboring a type III plasmid targeting its *cI*-like repressor gene. Following plaque re-isolations, a 791 bp deletion was confirmed by PCR and Sanger sequencing with primers JW443/JW444. The ɸNM1γ6α1 phage is a mutant of ɸNM1γ6 that was similarly isolated while infecting lawns harboring the pGG100 plasmid (gp32*), although the presence of a target site deletion was not confirmed after plaque re-isolations. ɸNM2γ1 is a clear mutant of ɸNM2 that was isolated during infection of lawns harboring the pGG152 type III plasmid targeting its *cI*-like repressor gene, although the presence of a target site deletion was not confirmed after plaque re-isolations. For homology-directed editing of phages, we used a variation of phage editing methods described previously^73,74^. Briefly, phages were propagated on hosts containing homology plasmids for recombination with phage genomes, and the resultant lysates were used to infect soft agar lawns containing a suitable CRISPR–Cas plasmid that can counter-select against parental genotypes. Homologies were designed such that recombinant phages would lack a target sequence for the type III CRISPR–Cas plasmid used in counter selection. The ɸNM4γ4α2 phage was created by replacing a 40 bp sequence containing the *gp32* spacer’s partially matched target in ɸNM4γ4 with a perfectly matched target sequence for the *nes* spacer^4^. ɸNM4γ4 was first propagated on lawns of RN4220 containing the homology plasmid, pAV43, and recombinants were subsequently selected during infection of lawns containing the pGG12^29^ CRISPR–Cas plasmid. After an additional round of counter selection with pGG12, a single ɸNM4γ4α2 plaque was isolated, and the presence of its recombinant target was confirmed by Sanger sequencing of PCR products generated with primers oGG38/oGG40. The ɸNM1-Erm^R2^ phage was constructed similarly by first propagating the ɸNM1-Erm^R^ phage^29^ on lawns containing the pGG90 homology plasmid, which includes the *tyc-1* allele SNP in the 5′ UTR of *ermC*, and subsequently selecting for recombinants lacking a nearby 182 bp fragment that encompasses the target for the pGG91 CRISPR–Cas plasmid. The presence of the recombinant sequences in ɸNM1-Erm^R2^ was confirmed by PCR and Sanger sequencing with primers oGG192/oGG191.

### Transduction

Subcultures were grown at 37 °C in TSB supplemented with CaCl_2_ at 5 mM for 1 h 25 min (~0.5–1.0 attenuance, *D*_600 nm_), and 990 µl aliquots were subsequently mixed with 10 µl of transducing lysate containing the plasmid of interest in autoclaved 1.5 ml tubes (Eppendorf). After 15 min of growth at 37 °C, infected cultures were treated with filter-sterilized sodium citrate to a final concentration of 40 mM, and then pelleted by centrifugation at 16,100 *× g* for 2 min with refrigeration (4 °C). When transducing pE194 or pT181 plasmids, an additional 1 h 45 min of growth was allowed immediately after treatment with citrate and prior to centrifugation. Following centrifugation, fresh TSB supplemented with sodium citrate (40 mM) was used to resuspend and dilute pellets for plating on solid media supplemented with both sodium citrate at 20 mM and antibiotics to select for the transductants. To avoid residual free phage, an additional re-streak was performed on solid media in the presence of citrate (20 mM) and antibiotics to select for the strain’s plasmid(s).

### Phage-sensitivity assay

Throughout cloning and strain construction procedures, or as otherwise needed, clones were readily checked for sensitivity to a phage of interest according to the streak method previously performed with ɸNM2^29^, except that “clear” mutant (lytic) derivatives described above were used in place of their parental temperate phages to facilitate scoring, and chloramphenicol-supplemented TSA without added CaCl_2_ was usually used in place of HIA.

### Strain construction

Single lysogens of TB4 were obtained by mixing 100 µl of a TB4 overnight culture with either ɸNM1 or ɸNM4 (MOI ~ 0.1–10) in HIA soft agar supplemented with CaCl_2_ at 5 mM, incubating overnight at 37 °C, and re-streaking from the resulting turbid lysate lawns. After an additional re-streak from single colonies, a clone was selected for each, and immunity groups were confirmed by assaying for sensitivity to clear mutants (ɸNM1γ6^29^ or ɸNM4γ4^24^). Both clones were also typed via PCR using ɸNM1- or ɸNM4-specific primer pairs, oGG6/oGG7 or oGG10/oGG11, respectively. The TB4::ɸNM4+ɸNM1 double lysogen was constructed and typed similarly, except that an overnight culture of TB4::ɸNM4 was mixed with ɸNM1 in soft agar to produce the turbid lysate lawn. ɸNM1 prophage mutants were constructed via CRISPR-assisted genome editing as outlined below. All plasmid-containing lysogenic derivatives were subsequently generated via transduction. Plasmid-containing non-lysogens were generated either via transformation of electrocompetent TB4 cells, or via transduction. Where applicable, strains were always marked via transduction with pE194 or pT181 plasmids first, followed by delivery of the CRISPR–Cas plasmid in a subsequent round of transduction.

### CRISPR-assisted genome editing of *S. aureus*

Homology-directed allelic exchange coupled with CRISPR–Cas counter selection was performed essentially as described previously for the pWJ327 allelic replacement system^75^, with modifications. pWJ327-derived vectors containing homology to ɸNM1 and the prophage mutation of interest were used to transform electrocompetent RN4220 non-lysogens instead of RN4220::Φ12, and a subsequent transduction step was used to transfer the plasmids from RN4220 to TB4::ΦNM1. Transduction was performed with ΦNM4γ4 as described above, except that transducing lysates were raised and injected at 28 °C, with 2 h 30 min of growth allowed for the recipient subculture. After isolating putative co-integrants via two consecutive re-streaks at 37 °C in the presence of chloramphenicol and verifying them with PCR using primers W1250/oGG7 (pGG170ts) or W1250/oGG32 (pGG172ts), clones were inoculated overnight in plain media at 37 °C instead of 28 °C. Subcultures were grown at 28 °C in plain media supplemented with CaCl_2_ at 5 mM to obtain logarithmic-phase cultures for treatment with the pWJ326 phagemid, but treated cultures were plated at 30 °C on media containing erythromycin after 3 h of growth at 30 °C rather than 1 h. An additional re-streak at 30 °C was performed in the presence of erythromycin before clones were checked by PCR and Sanger sequencing with primers oGG455/oGG470 (pGG170ts) or oGG471/oGG474 (pGG172ts) for the presence of the desired mutation (ɸNM1^P*cro*^ or ɸNM1^*ind−*^, respectively). Clones which had also lost the integrated pWJ327 amplicon (again determined by PCR with primers W1250/oGG7 or W1250/oGG32) were inoculated overnight in plain media but grown at 42 °C instead of 37 °C in order to cure the strain of pWJ326. Dilutions were plated on plain media at 37 °C, and single colonies were replica-plated at 37 °C on both plain and erythromycin-supplemented media to confirm plasmid loss. After re-streaking an erythromycin-sensitive clone on plain media, a PCR amplicon spanning the entire homology region was procured with primers oGG33/oGG7 (pGG170ts) or oGG475/oGG32 (pGG172ts), and sequenced by Sanger using additional primers listed in Supplementary Table 4. The clone was also re-streaked on media supplemented with chloramphenicol as a final check for sensitivity to the antibiotic; and, checked for sensitivity to ΦNM1γ6 to ensure that lysogenic immunity had not been lost. The pGG170ts- and pGG172ts-derived strains were renamed GWG7 and GWG9, respectively. TSB and TSA media were used in place of BHI throughout. The pWJ326 phagemid lysate used in this work was procured using the method described previously^75^.

### Pairwise competition assays with marked lysogens

Aliquots of overnight cultures were mixed 1:1 by volume and then diluted for passaging and selective plating on tetracycline (5 µg/ml) and chloramphenicol or erythromycin (5 µg/ml) and chloramphenicol. Passaging and plating was repeated in this manner every 24 h for 6 days, and relative frequencies were calculated at each interval as the number of Tet^R^+Cm^R^ colony forming units (CFU) divided by the sum of Erm^R^+Cm^R^ CFU plus Tet^R^+Cm^R^ CFU.

### Enumeration of plaque-forming units from lysogenic cultures

“Spontaneous” particle release from single lysogens was measured essentially as described previously^29^, except that filtered supernatants were collected immediately after 12 h of growth overnight. For quantification of particles released from MMC-induced cultures, overnight cultures were grown for 15 h and subcultures were grown for 1 h 15 min (~ 0.3–0.6 attenuance, *D*_600 nm_) before treatment with MMC. Following 4 h of growth post treatment, subcultures were pelleted by centrifugation at 4303 ×* g* for 6 min, and filtered supernatants were collected for serial dilution and spotting on lawns of TB4 harboring the CRISPR–Cas parent vector.

### High-resolution growth curves

Overnight cultures grown for 14 h were subcultured essentially as described above, except that 200 µl culture volumes were used in 96-well microplates, and incubation at 37 °C with shaking was performed with an Infinite M200 PRO plate reader (TECAN) measuring attenuance (*D*_600 nm_) every 10 min. In prophage induction experiments, subcultures were treated with MMC to a final concentration of 2.0 µg/ml, following 70 min of growth. In phage infection experiments, subcultures were treated with ɸNM1γ6 at a multiplicity of infection ~7.5, following 45 min of growth. For each overnight culture, an additional subculture was performed in parallel to monitor growth in the absence of MMC or ɸNM1γ6 treatment.

### Efficiency of plaquing

Assays were performed by spotting high-titer lysate serial dilutions onto both CRISPR-containing and control lawns to enumerate pfu and calculate efficiency as pfu on CRISPR-containing cells/pfu on control cells, as described previously^29^, except that the sensitive control lawns utilized a TB4 derivative harboring the pGG79 parent vector.

### Lysogenization with *ermC*-marked ΦNM1

Lysogenization was performed essentially as described previously^29^, except that overnight cultures were always grown for 14 h, TSB broth was used in place of HIB, multiplicity of infection was ~1, and incubation times were reduced to 10 min on ice and 20 min at 37 °C. Following the 20 min incubation, cultures were supplemented and plated with citrate as described for the transduction protocol above, except that chloramphenicol was maintained in all media and plates were also supplemented with erythromycin (5 µg/ml) to quantify acquisition of the marked prophage. The ΦNM1-Erm^R2^ phage used for infections is a variant of ΦNM1-Erm^R29^ with a modified *ermC* 5′ UTR that includes the SNP found in the *tyc-1* allele, previously reported to improve constitutive expression of the cassette’s gene product in *B. subtilis* ^76^. To estimate the concentration of total recipients harboring CRISPR–Cas systems, serial dilutions of the untreated overnight cultures were plated in the presence of chloramphenicol alone.

### Batch competition of unmarked lysogens for deep sequencing

Aliquots of overnight cultures were mixed in equal proportions by volume and then diluted for passaging as described above. The remaining culture (~2 ml) was pelleted by centrifugation at 4303 × *g* for 6 min. Supernatants were discarded, and pellets were stored at −20 °C after drying for 10 min at benchtop. Passaging and storage of pellets was repeated in this manner every 24 h for 3 days.

### Preparation of DNA for deep sequencing

Frozen pellets from batch competition experiments were thawed on ice for 20 min and then miniprepped as described above. In total, 100 ng of miniprepped DNA from each sample was used as the template for barcoded PCRs to amplify a region of the CRISPR array that fully spans spacer position 2, using forward and reverse primers with identical barcode pairs. Barcoded PCR products were gel purified and then pooled at roughly equal proportions by normalizing to the least-concentrated NanoDrop Spectrophotometer (Thermo) reading. Library preparation was subsequently carried out on the pooled sample using a TruSeq Nano DNA LT kit (Illumina).

### Deep sequencing and data analysis

Deep sequencing was performed on an Illumina MiSeq essentially as described in the manufacturer’s protocol for low-complexity amplicon sequencing with the v3 reagent kit (~25% PhiX spike-in). Fastq output files were parsed using the Biopython package for python, and reads containing the expected sequences with 100% identity (either forward or reverse) were tallied for each barcode set. Relative frequency datapoints are plotted as the number of reads containing a particular barcode and the perfect or partially matching spacer of interest divided by the sum of that count plus reads containing the fully mismatched spacer with the same barcode.

### Pairwise competition assays with marked non-lysogens

Aliquots of overnight cultures grown for 14 h were mixed 1:1 by volume and then subcultured for 45 min, prior to withdrawing an aliquot for selective plating on tetracycline (5 µg/ml) and chloramphenicol or erythromycin (5 µg/ml) and chloramphenicol, and subsequently infecting the remaining culture with ɸNM1γ6 at a multiplicity of infection ~7.5. Following 23 h and 15 min of infection at 37 °C, cultures were treated with filter-sterilized sodium citrate to a final concentration of 40 mM, diluted in similarly supplemented media, and then grown on plates containing tetracycline (5 µg/ml) and chloramphenicol or erythromycin (5 µg/ml) and chloramphenicol—in each case also supplemented with sodium citrate (20 mM). Relative frequencies were calculated at each interval as the number of Tet^R^+Cm^R^ CFU divided by the sum of Erm^R^+Cm^R^ CFU plus Tet^R^+Cm^R^ CFU.

### Statistical analyses

Student’s *t* test and ANOVA analyses were performed using the stats package in R (version 3.4.0), and lme analyses were performed using the afex package in R. Statistical methods were not used to predetermine sample sizes prior to experimentation.

### BLAST-assisted search for self-matching spacers in Staphylococci

The 15 fully sequenced staphylococcal genomes previously determined to contain type III-A systems^49^ were analyzed further for the presence of CRISPR spacers that co-occur with one or more target sequences in the same genome. To search for perfectly matching targets, a FASTA-formatted master list containing their 70 unique spacers was aligned to each genome using NCBI’s online megablast tool, and results were manually screened for the presence or absence of spacer hits that matched to both a CRISPR locus and at least one additional region elsewhere in the genome. Default parameters (August 2017) were used, except that the filter for low-complexity regions was left unchecked. To facilitate the detection of partially mismatched spacers, alignments were repeated using NCBI’s online discontinuous megablast tool. Again, default parameters were used, except that template length was set to “16”, template type was set to “two templates”, and the filter for low-complexity regions was left unchecked. In order to compile the 70-spacer master list, the previously available^49^ collection of 39 unique spacers from *S. aureus* and *S. argenteus* type III-A systems was expanded to include spacers from the non-*aureus* and non-*argenteus* Staphylococci in this cohort. The additional spacers were manually collected from sequences located between previously defined type III-A repeats^49^, except for the downstream-most spacer in each CRISPR array, which was instead defined as the sequence located between a known repeat and a degenerate repeat. The sequence of each degenerate repeat was manually inferred based on its distal location in the CRISPR array, partial homology to the nearest upstream repeat, and length (~36 bp). Spacers identified in this manner were subsequently added to the master list, if not already present within the file.

### Data availability

Relevant data supporting the findings of the study are available in this published article and its Supplementary Information files, and from the corresponding author upon request.

## Electronic supplementary material

Supplementary Information
