# Supplementary material for: Incomplete prophage tolerance by type III-A CRISPR-Cas systems reduces the fitness of lysogenic hosts
Source: Nat Commun. 2018 Jan 4;9:61. doi: 10.1038/s41467-017-02557-2 (PMC5754349; doi:10.1038/s41467-017-02557-2)
Supplement: Supplementary file 1 — Supplementary Information [file 41467_2017_2557_MOESM1_ESM.pdf]

Supplementary Information

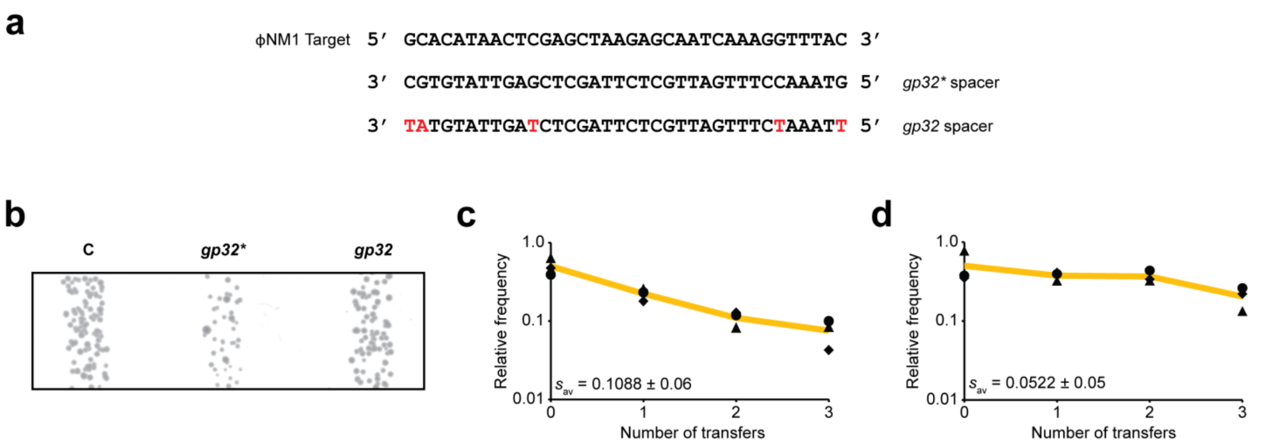

Supplementary Figure 1. Maintenance of conditionally tolerant type III-A CRISPR-Cas systems in lysogenic hosts can incur fitness costs. (**a**) Sequence summary of the perfectly matched *gp32* spacer (*gp32**) and the originally identified *gp32* spacer with 5 mismatches (*gp32*), relative to their target in ΦNM1. Red lettering denotes the position of mismatches. (**b**) Colony size comparison of Tet^R-marked TB4::ΦNM1 single lysogens harboring CRISPR-Cas plasmids with either non-targeting spacers alone (C), the *gp32** spacer, or the *gp32* spacer. Picture is representative of a biological replicate for each strain plated in the presence of chloramphenicol to select for the CRISPR-Cas plasmids. (**c–d**) Pairwise competition experiments for the *gp32** (c) or *gp32* (d) spacers that license conditional tolerance of ΦNM1. In each case, an Erm^R-marked control lysogen harboring the parent vector with non-targeting spacers was competed against a Tet^R-marked conditionally tolerant lysogen. Relative frequencies (y-axis) are plotted against the number of transfers (x-axis), with one transfer per day. Individual values from each biological replicate (*n* = 3) are depicted in black as a triangle, circle, or rhombus. Solid lines represent the average change in relative frequency across the three replicates, and share color coding with the target gene highlighted in Figure 1a. The s_{av} values are derived from Supplementary Table 1.

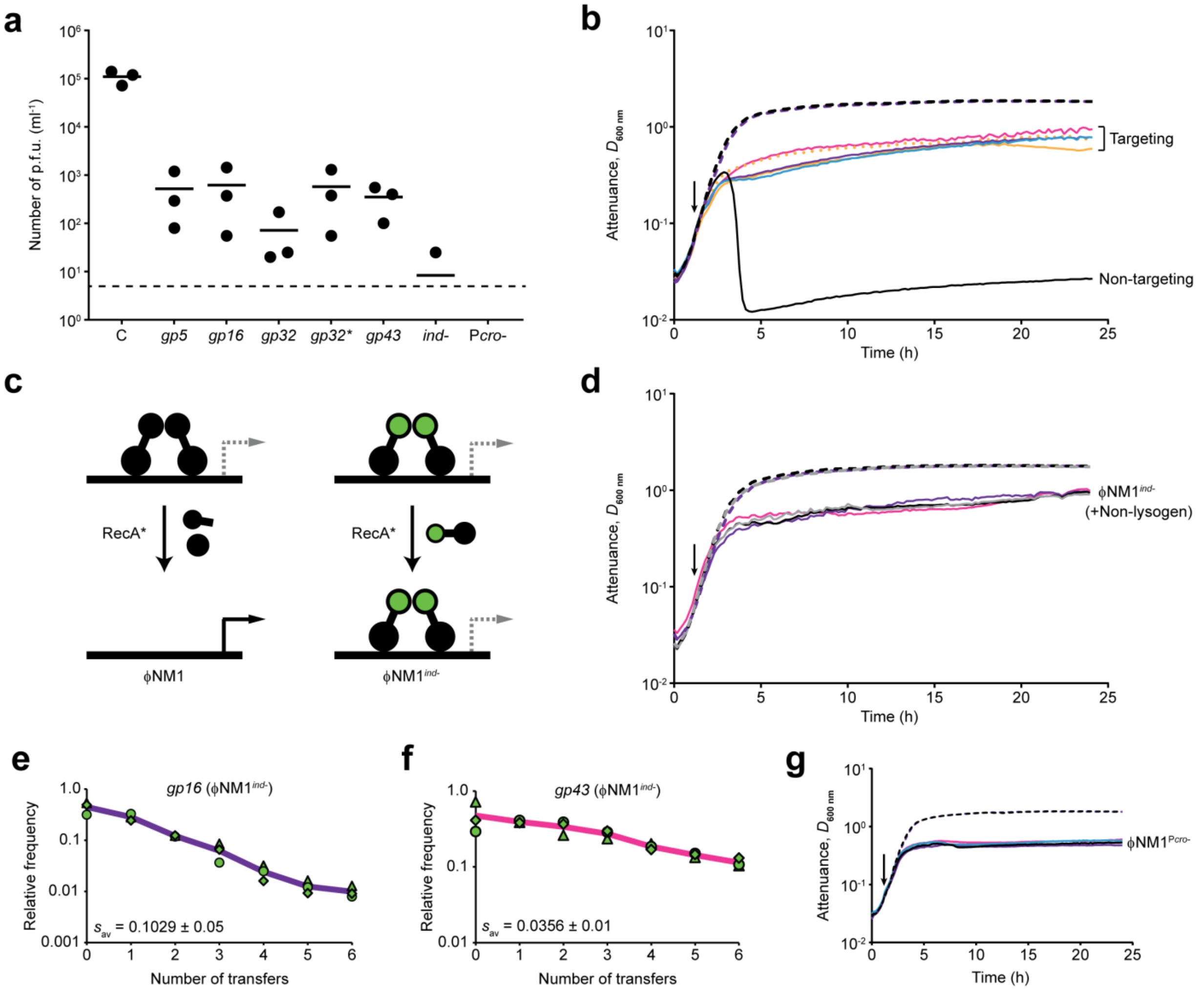

Supplementary Figure 2. Prophage inducibility is not a prerequisite for incomplete tolerance by type III-A systems. (**a**) Quantification of plaque forming unit concentrations (plaque-forming units per millilitre) in filtered supernatants from overnight cultures of Tet^{R}-marked TB4::ΦNM1 single lysogens harboring CRISPR-Cas plasmids with one of several targeting spacers, or the control parent vector with non-targeting spacers (C). Horizontal bars indicate the mean value of n = 3 biological replicates, plotted as black dots in each column. Tet^{R}-marked lysogens harboring the control parent vector and mutant prophages ΦNM1^{ind-} or ΦNM1^{Pcro-} were also tested, and plaques were only detected in one of the replicates for ΦNM1^{ind-}. Dotted line represents the limit of detection under these assay conditions. (**b**) High-resolution growth curves of Tet^{R}-marked TB4::ΦNM1 single lysogens harboring the CRISPR-Cas plasmids tested in panel '**a**'. Solid lines represent the average attenuance ($D_{600\ nm}$) measured from n = 3 biological replicates with Mitomycin C (MMC) added at the indicated time point (black arrow), and are colored to match the target ORFs highlighted in Figure 1a or black for the control parent vector. A dotted line is used for the *gp32* (mismatched) spacer, but note that individual dots do not correspond to measurements taken every 10 minutes.

For comparison, average attenuance measured from $n = 3$ biological replicates in the absence of MMC is plotted for two strains (dashed lines). (**c**) Schematic diagram illustrating different outcomes expected for the wild type ΦNM1 prophage (left) or the ΦNM1^{ind-} prophage mutant (right) in response to a canonical SOS-inducing signal. Monomers of *cI*-like repressor are depicted as black bar bells, with green spots for the ΦNM1^{ind-} mutant indicating the presence of a S124A substitution in the C-terminal serine protease domain. When intact repressors are present, only leaky transcription originates from the regulated promoter (dashed grey bent arrows). Under inducing conditions, autoproteolysis of wild type repressor is stimulated by the presence of activated RecA (RecA*) and allows transcription required for the lytic cycle (solid black bent arrow), while ΦNM1^{ind-} mutant repressor remains intact and continues to allow only leaky transcription. (**d**) High-resolution growth curves as in panel ‘b’, except that Tet^{R}-marked single lysogens harbor the ΦNM1^{ind-} mutant prophage. A subset of the CRISPR-Cas plasmids was tested, as indicated by color-coding; a Tet^{R}-marked TB4 non-lysogen harboring the control parent vector was also tested (grey lines). (**e–f**) Pairwise competition experiments as in Figure 1c–d, except that targeting and control lysogens both contain the ΦNM1^{ind-} prophage mutations. (**g**) High-resolution growth curves as in panel ‘b’, except that Tet^{R}-marked single lysogens harbor the ΦNM1^{Pcro-} mutant prophage. A subset of the CRISPR-Cas plasmids was tested, as indicated by color-coding.

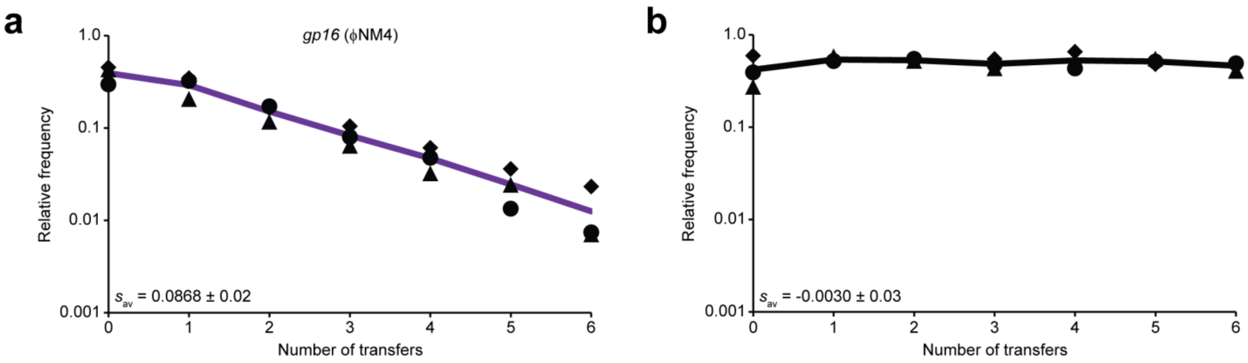

Supplementary Figure 3. Detection of fitness costs associated with conditional tolerance in TB4::ΦNM4 single lysogens. (**a**) Pairwise competition experiment with marked TB4::ΦNM4 single lysogens. An Erm^R-marked control lysogen harboring the parent vector with non-targeting spacers was competed against a Tet^R-marked conditionally tolerant lysogen. Relative frequencies (y-axis) are plotted against the number of transfers (x-axis), with one transfer per day. Individual values from each biological replicate (n = 3) are depicted in black as a triangle, circle, or rhombus. Solid line represents the average change in relative frequency across the three replicates, and shares color coding with the target gene highlighted in Figure 2a. The s_{av} value is derived from Supplementary Table 1. (**b**) Control co-culture experiment. Pairwise competition was performed as in ‘a’ above, except that differentially-marked TB4::ΦNM4 lysogens both harbor the same parent vector with non-targeting spacers.

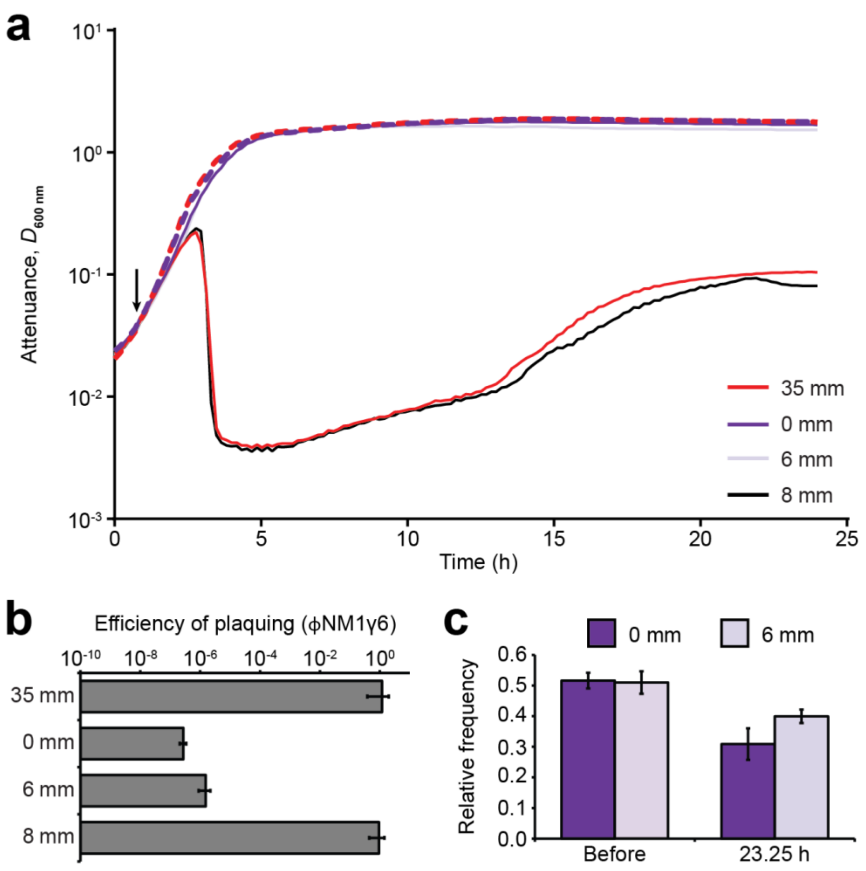

Supplementary Figure 4. Protectiveness of the 0- and 6-mismatched *gp16* variants is comparable during lytic infections. (**a**) High-resolution growth curves of Tet^R-marked TB4 non-lysogens harboring the *gp16* CRISPR-Cas plasmid or one of its mismatched (mm) variants. Solid lines represent the average attenuance ($D_{600\ nm}$) measured from *n* = 3 biological replicates with the virulent phage, ϕNM1γ6 (MOI ~7.5), added at the indicated time point (black arrow), and are color-coded according to the labeled inset. For comparison, average attenuance measured from *n* = 3 biological replicates in the absence of ϕNM1γ6 is plotted for the 0- and 35-mismatched strains (dashed lines). (**b**) Plaquing efficiency of ΦNM1γ6 on lawns of the Tet^R-marked TB4 non-lysogens tested in panel 'a', as indicated. Efficiency ratios were calculated for each plasmid relative to plaques quantified on a TB4 lawn harboring the non-targeting parent vector. Error bars, mean ± s.d. (*n* = 3, technical replicates). (**c**) Pairwise co-culture experiment where an Erm^R-marked TB4 non-lysogen with the *gp16* CRISPR-Cas plasmid was competed against a Tet^R-marked TB4 non-lysogen harboring either the same *gp16* CRISPR-Cas plasmid or the 6-mismatched variant, in the presence of ΦNM1γ6 (MOI ~7.5). Relative frequencies (y-axis) detected immediately before or 23.25 hours after infection with ΦNM1γ6 are plotted for each of the Tet^R-marked strains tested. Error bars, mean ± s.d. (*n* = 3, biological replicates).

Supplementary Table 1. Selection coefficients determined in fitness assays.

Prophage(s)	Spacer[(a)]	Figure	Mean *s*[(b)]	s.d.[(c)]	Median *s*
ΦNM1	*gp32**	S1c	0.1088	0.0561 (*n* = 3)	0.1162
ΦNM1	*gp32* [5 mm]	S1d	0.0522	0.0476 (*n* = 3)	0.0530
ΦNM1	*gp5*	1b	0.1036	0.0394 (*n* = 6)	0.1062
ΦNM1	*gp16*	1c	0.0822	0.0663 (*n* = 6)	0.0680
ΦNM1	*gp43*	1d	0.0327	0.0283 (*n* = 6)	0.0192
ΦNM1	Parent vector	1e	0.0077	0.0344 (*n* = 6)	0.0005
ΦNM1[Pcro-]	*gp5*	1g	0.0137	0.0256 (*n* = 6)	0.0168
ΦNM1[Pcro-]	*gp16*	1h	0.0531	0.0098 (*n* = 6)	0.0557
ΦNM1[Pcro-]	*gp43*	1i	0.0334	0.0262 (*n* = 6)	0.0373
ΦNM1[ind-]	*gp16*	S2e	0.1029	0.0498 (*n* = 6)	0.0981
ΦNM1[ind-]	*gp43*	S2f	0.0356	0.0118 (*n* = 6)	0.0335
ΦNM1	*gp16* (*csm3*)	2b	0.0745	0.0504 (*n* = 6)	0.0844
ΦNM1	*gp16* (*cas10*)	2c	0.0055	0.0170 (*n* = 6)	0.0083
ΦNM1	*gp16* (*csm6*)	2d	0.0113	0.0177 (*n* = 6)	0.0088
ΦNM1	*gp16* (*csm3/csm6*)	2e	0.0258	0.0189 (*n* = 6)	0.0233
ΦNM4+ΦNM1	*gp5*	3e	0.1059	0.0441 (*n* = 3)	0.1288
ΦNM4+ΦNM1	*gp16*	3e	0.3341	0.0775 (*n* = 3)	0.3396
ΦNM4+ΦNM1	*gp43*	3e	0.0581	0.0528 (*n* = 3)	0.0300
ΦNM1	*gp16*	4c	0.1080	0.0404 (*n* = 3)	0.1076
ΦNM1	*gp16* [2 mm]	4c	0.0722	0.0320 (*n* = 3)	0.0689
ΦNM1	*gp16* [4 mm]	4c	0.0115	0.0474 (*n* = 3)	0.0198
ΦNM1	*gp16* [6 mm]	4c	0.0268	0.0163 (*n* = 3)	0.0210
ΦNM1	*gp16* [8 mm]	4c	-0.0191	0.0355 (*n* = 3)	-0.0133
ΦNM1	*gp16* [10 mm]	4c	0.0002	0.0111 (*n* = 3)	0.0003
ΦNM1	*gp16* [12 mm]	4c	0.0038	0.0147 (*n* = 3)	0.0110
ΦNM1	*gp16* [14 mm]	4c	-0.0206	0.0393 (*n* = 3)	-0.0122
ΦNM1	*gp16* [16 mm]	4c	0.0056	0.0139 (*n* = 3)	0.0115
ΦNM4+ΦNM1	*gp16*	4d	0.2155	0.0780 (*n* = 3)	0.1902
ΦNM4+ΦNM1	*gp16* [2 mm]	4d	0.2090	0.0289 (*n* = 3)	0.1949
ΦNM4+ΦNM1	*gp16* [4 mm]	4d	0.0812	0.0224 (*n* = 3)	0.0939
ΦNM4+ΦNM1	*gp16* [6 mm]	4d	0.0494	0.0238 (*n* = 3)	0.0604
ΦNM4+ΦNM1	*gp16* [8 mm]	4d	-0.0192	0.0201 (*n* = 3)	-0.0098
ΦNM4+ΦNM1	*gp16* [10 mm]	4d	-0.0089	0.0043 (*n* = 3)	-0.0090
ΦNM4+ΦNM1	*gp16* [12 mm]	4d	0.0201	0.0604 (*n* = 3)	-0.0051
ΦNM4+ΦNM1	*gp16* [14 mm]	4d	-0.0212	0.0216 (*n* = 3)	-0.0181
ΦNM4+ΦNM1	*gp16* [16 mm]	4d	-0.0145	0.0139 (*n* = 3)	-0.0117
ΦNM4	*gp16*	S3a	0.0868	0.0209 (*n* = 6)	0.0919
ΦNM4	Parent vector	S3b	-0.0030	0.0262 (*n* = 6)	0.0045

(a) For spacers possessing mismatches relative to their prophage target, the number of mismatches (mm) is written in brackets. For spacers present in CRISPR-Cas plasmids with one or more mutant *cas* genes, the mutated gene is written in parentheses.

(b) Selection coefficients, '*s*', determined from the change in relative frequency (or average relative frequency, where available) after each transfer interval in fitness assays. Values were extracted from the equation, $dq/dt = -q(1 - q)s$, where q is the relative frequency of the spacer being assayed, and t = 9.97 generations is assumed for 1:1000 dilutions at each transfer interval. Positive selection coefficients ($s > 0$) suggest that the spacer is associated with a fitness cost.

(c) Standard deviation.

Supplementary Table 2. Summary of statistical comparisons presented in this work.

Test	Datasets Compared[(a)]	*p*-value
Linear mixed effect model	S1c, S1d	0.0983
Linear mixed effect model	1e, 1b	0.0008
Linear mixed effect model	1e, 1c	0.0015
Linear mixed effect model	1e, 1d	0.0493
One-way ANOVA	1e*	0.6342
One-way ANOVA	1b, 1c, 1d	0.0499
Linear mixed effect model	S2e, 1c	0.9786
Linear mixed effect model	S2f, 1d	0.5431
Linear mixed effect model	1e, 1g	0.2721
Linear mixed effect model	1h, 1c	0.0321
Linear mixed effect model	1i, 1d	0.6711
Linear mixed effect model	2b, 1c	0.5655
Linear mixed effect model	1e, 2c	0.5341
Linear mixed effect model	2d, 1c	0.0026
Linear mixed effect model	2e, 1c	0.0272
One-way ANOVA	3b**	0.1906
One-sided *t* test	3e_*gp16*, 3e_*gp5*	0.0095
One-sided *t* test	3e_*gp16*, 3e_*gp43*	0.0049
One-sided *t* test	3e_*gp16*, 1c (0-3)	0.0056
One-sided *t* test	3e_*gp16*, S3a (0-3)	0.0095
Two-sided *t* test	3e_*gp5*, 1b (0-3)	0.6322
Two-sided *t* test	3e_*gp43*, 1d (0-3)	0.5643
Two-sided *t* test	S4c_0mm***, S4c_6mm***	0.0669
One-sided *t* test	4c_0mm, 4c_4mm	0.0280
One-sided *t* test	4c_0mm, 4c_6mm	0.0290
One-sided *t* test	4d_0mm, 4d_4mm	0.0436
One-sided *t* test	4d_0mm, 4d_6mm	0.0281
One-way ANOVA	1e (0-3), 4c_8mm, 4c_10mm, 4c_12mm, 4c_14mm, 4c_16mm	0.7408
One-way ANOVA	1e (0-3), 4d_8mm, 4d_10mm, 4d_12mm, 4d_14mm, 4d_16mm	0.6265
Two-sided *t* test	1c (0-3), 4c_0mm	0.3862
Two-sided *t* test	3e_*gp5*, 4d_4mm	0.4508
Two-sided *t* test	3e_*gp5*, 4d_6mm	0.1433

(a) Linear mixed effect model (lme) tests compared datasets with multiple samples (*n* = 3), each containing either 3 or 6 selection coefficient replicates (depending on the number of transfers carried out in the corresponding experiment). Unless otherwise noted, *t* tests compared the means of samples that each contained 3 selection coefficient replicates. Parenthesis indicates that selection coefficient data from a subset of the transfer intervals were included for comparison, and relative frequencies were averaged across samples within the dataset before calculating selection coefficients for each transfer interval. *, indicates that relative frequency values were directly compared over time within the dataset (with biological 3 replicates at each time point). **, denotes a comparison between Cm^R, Erm^R c.f.u. (ml^{-1}) values from lysogenization experiments with either the 35 mm, *gp5*, *gp16*, or *gp43* spacers. ***, indicates that the dataset is comprised of fold change values computed for relative frequency measurements before and after infection with ϕNM1γ6, from biological replicates testing the perfectly matching (0mm) or partially mismatched (6mm) spacer.

Supplementary Table 3. Spacers tested in this study.

Spacer[a]	Sequence (5'-3')[b]	Plasmid Name
Parent vector	TGAGACCAGTCTCGGAAGCTCAAAGGTCTC	pGG79
gp5	ATGTCTTAGCAATTCTAAAAGCATCTCTAGGTTTA	pGG126
gp16	TAGAACAGTCGCGCAGACTGATTTCTTCATAACCT	pGG102
gp32 [5 mm]	TTAAATCTTTGATTGCTCTTAGCTCTAGTTATGTAT	pGG122
*gp32**	GTAAACCTTTGATTGCTCTTAGCTCGAGTTATGTGC	pGG100
gp43	ATTCGTCATCTTCAAGTAATGCCTCTAAATCAATA	pGG165
gp16 [35 mm]	GCTCCACTGATATACTCAGTCGGGAGGACGCCAAG	pGG153
gp16 [2 mm]	TCGAACAGTCGCGCAGACTGATTTCTTCATAACAT	pGG154
gp16 [4 mm]	TCGCACAGTCGCGCAGACTGATTTCTTCATACCAT	pGG155
gp16 [6 mm]	TCGCAAAGTCGCGCAGACTGATTTCTTCAGACCAT	pGG156
gp16 [8 mm]	TCGCAAATTCGCGCAGACTGATTTCTTAAGACCAT	pGG157
gp16 [10 mm]	TCGCAAATTAGCGCAGACTGATTTCGTAAGACCAT	pGG158
gp16 [12 mm]	TCGCAAATTAGAGCAGACTGATTGCGTAAGACCAT	pGG160
gp16 [14 mm]	TCGCAAATTAGAGAAGACTGAGTGCGTAAGACCAT	pGG161
gp16 [16 mm]	TCGCAAATTAGAGAATACTTAGTGCGTAAGACCAT	pGG162

(a) '*gp'* numbers correspond to open reading frames with a matching target sequence in ΦNM1 (Accession: NC_008583.1). Mismatched variants are also listed, with the number of mismatches (mm) denoted within brackets. Each spacer is located between repeats 2 and 3 of the CRISPR array. A placeholder sequence with two opposing BsaI restriction sites is present at this position in the parent vector.

(b) In order to avoid excessive matching between the 5' crRNA tag and the target's corresponding flanking sequence, which has been shown to abrogate DNA targeting by the type III-A system[1,2], spacer-target combinations were selected such that no more than 1 bp of complementarity was present at flanking sequences in each case.

Supplementary Table 4. Oligonucleotides used in this study.

Name	Sequence	Primary Purpose
**oGG281**	GTATCGATCGAGACCTTTGAGCTTCCGAGAC	Construction of pGG79 control/parent vector
**oGG282**	CCACCCCGAAGAAAAGGGGACGAGAACTAAATCTAACAACACTCTAAAAAATTG	Construction of pGG79 control/parent vector
**oGG330**	GAACGTAAACCTTTGATTGCTCTTAGCTCGAGTTATGTGCG	Construction of pGG100
**oGG331**	CGATCGCACATAACTCGAGCTAAGAGCAATCAAAGGTTTAC	Construction of pGG100
**oGG332**	GAACTAGAACAGTCGCGCAGACTGATTTCTTCATAACCTG	Construction of pGG102
**oGG333**	CGATCAGGTTATGAAGAAATCAGTCTGCGCGACTGTTCTA	Construction of pGG102
**oGG385**	GAACTTAAATCTTTGATTGCTCTTAGCTCTAGTTATGTATG	Construction of pGG122
**oGG386**	CGATCATACATAACTAGAGCTAAGAGCAATCAAAGATTTAA	Construction of pGG122
**oGG397**	GAACATGTCTTAGCAATTCTAAAAGCATCTCTAGGTTTAG	Construction of pGG126
**oGG398**	CGATCTAAACCTAGAGATGCTTTTAGAATTGCTAAGACAT	Construction of pGG126
**oGG435**	GAACGCTCCACTGATATACTCAGTCGGGAGGACGCCAAGG	Construction of pGG153
**oGG436**	CGATCCTTGGCGTCCTCCCGACTGAGTATATCAGTGGAGC	Construction of pGG153
**oGG437**	GAACTCGAACAGTCGCGCAGACTGATTTCTTCATAACATG	Construction of pGG154
**oGG438**	CGATCATGTTATGAAGAAATCAGTCTGCGCGACTGTTCGA	Construction of pGG154
**oGG439**	GAACTCGCACAGTCGCGCAGACTGATTTCTTCATACCATG	Construction of pGG155
**oGG440**	CGATCATGGTATGAAGAAATCAGTCTGCGCGACTGTGCGA	Construction of pGG155
**oGG441**	GAACTCGCAAAGTCGCGCAGACTGATTTCTTCAGACCATG	Construction of pGG156
**oGG442**	CGATCATGGTCTGAAGAAATCAGTCTGCGCGACTTTGCGA	Construction of pGG156
**oGG443**	GAACTCGCAAATTCGCGCAGACTGATTTCTTAAGACCATG	Construction of pGG157
**oGG444**	CGATCATGGTCTTAAGAAATCAGTCTGCGCGAATTTGCGA	Construction of pGG157
**oGG445**	GAACTCGCAAATTAGCGCAGACTGATTTCGTAAGACCATG	Construction of pGG158
**oGG446**	CGATCATGGTCTTACGAAATCAGTCTGCGCTAATTTGCGA	Construction of pGG158
**oGG447**	GAACTCGCAAATTAGAGCAGACTGATTGCGTAAGACCATG	Construction of pGG160
**oGG448**	CGATCATGGTCTTACGCAATCAGTCTGCTCTAATTTGCGA	Construction of pGG160
**oGG449**	GAACTCGCAAATTAGAGAAGACTGAGTGCGTAAGACCATG	Construction of pGG161
**oGG450**	CGATCATGGTCTTACGCACTCAGTCTTCTCTAATTTGCGA	Construction of pGG161
**oGG451**	GAACTCGCAAATTAGAGAATACTTAGTGCGTAAGACCATG	Construction of pGG162
**oGG452**	CGATCATGGTCTTACGCACTAAGTATTCTCTAATTTGCGA	Construction of pGG162
**oGG461**	GAACATTCGTCATCTTCAAGTAATGCCTCTAAATCAATAG	Construction of pGG165
**oGG462**	CGATCTATTGATTTAGAGGCATTACTTGAAGATGACGAAT	Construction of pGG165
**oGG320**	GAACGTTTTGGTCGTAGAGCACACGGTTTAACGACTTAAG	Construction of pGG91
**oGG321**	CGATCTTAAGTCGTTAAACCGTGTGCTCTACGACCAAAAC	Construction of pGG91
**oGG432**	GAACCCAGTTGCACCACATGCAATATACGATACTAGTTTG	Construction of pGG152
**oGG433**	CGATCAAACTAGTATCGTATATTGCATGTGGTGCAACTGG	Construction of pGG152
**L6**	AAAGGTACCAAATTTAATGCTATTTTCCTTCGC	Type III CRISPR array sequence verification
**L50**	AAAAGATCTAATAATGTATTTACGCTGGGGC	Type III CRISPR array sequence verification
**PS153**	GGTAAATCAAAACTAACTAACAAATACATTAGTTTCCCACCTCTATCATC	Construction of pPS95
**PS154**	GATGATAGAGGTGGGAAACTAATGTATTTGTTAGTTAGTTTTGATTTACC	Construction of pPS95
**PS465**	GAATCTAGTATGATTGGAGCAATTGCTTCTCCTGTAGTTAGAGATTTGCAAACC	Construction of pPS95
**PS466**	GGTTTGCAAATCTCTAACTACAGGAGAAGCAATTGCTCCAATCATACTAGATTC	Construction of pPS95
**W852**	CCAACAAACGACTTTTAGTATAACC	Construction of other mutant type III plasmids
**W614**	GGTTATACTAAAAGTCGTTTGTTGG	Construction of other mutant type III plasmids
**PS565**	GTTAGAGTATTATATTTATCAAATAAGGGAG	Construction of pGG99 and pGG89
**PS566**	CTCCCTTATTTGATAAATATAATACTCTAAC	Construction of pGG99 and pGG89
**oGG457**	GAGCACAAATTAAGTATTAAAGAAG	Construction of pGG167
**oGG458**	CTTCTTTAATACTTAATTTGTGCTC	Construction of pGG167
**oGG424**	CATATTGCCTGATGAAGTGAATAG	Construction of pGG139
**oGG425**	CTATTCACTTCATCAGGCAATATG	Construction of pGG139
**oGG270**	TAAAATGTGGTTTGACAAACGAAAATTGGATAAAGTGGG	Additional type III plasmid sequencing
**L36**	AAAGGTACCTTATTACTACCTAAGATGATAGAGG	Additional type III plasmid sequencing
**W14**	ATCAATTTTTGTCCCAATTTTCAG	Additional type III plasmid sequencing
**W15**	CAAATTACTGCTATATATTCAGGC	Additional type III plasmid sequencing
**W16**	TTAAATTTTATTATGAAGCAGGACG	Additional type III plasmid sequencing
**W19**	CTACTTTAATAATTGAAAAAGATGG	Additional type III plasmid sequencing
**PS566**	CTCCCTTATTTGATAAATATAATACTCTAAC	Additional type III plasmid sequencing
**W125**	ATTTAGCGTCTATAGTAAAGGTG	Construction of pAV71
**PS557**	CCATGCACCGATTAAAAATAAAGCGGCACCGCCTGAATATATAGCAG	Construction of pAV71
**PS556**	CTGCTATATATTCAGGCGGTGCCGCTTTATTTTTAATCGGTGCATGG	Construction of pAV71
**W762**	CAAATCACCTTTACTATAGACGC	Construction of pAV71
**W1169**	CCGATTAAAAATAAAGCTGCACCGCCTGAATATATAGCAGTAATTTG	Construction of pWJ291
**W1170**	TATTCAGGCGGTGCAGCTTTATTTTTAATCGGTGCATGGGATG	Construction of pWJ291
**JW398**	GAACGTCTCATAATTTGAAATAGTAGCTTTTTTAGTGTT	Construction of JW233's type III plasmid
**JW399**	TTTAAACACTAAAAAAGCTACTATTTCAAATTATGAGAC	Construction of JW233's type III plasmid
**JW443**	CTTTAGGTAACTCATAAGTGAATGGTTG	Sequencing of the ϕ11γ2 deletion
**JW444**	GCCACTCTGTTAAATCAGTAACTTTG	Sequencing of the ϕ11γ2 deletion
**oGG387**	GTGTTGAACTTGAACAAAGTCAC	Sequencing of the ϕ11γ2β1 deletion

**oGG388**	TCATCAACTTTTATCCACGAGTC	Sequencing of the ϕ11γ2β1 deletion
**AV205**	ATACAAAACCACATACCTATCAACGTGATGAGCTTATTGGGG	Construction of pAV43
**AV202**	GAAGTATATAAATCATCAGTACAAAGGTTTACGTCCTGTTGAATCTTTG	Construction of pAV43
**AV203**	GATGATTTATATACTTCGGCATACGTAGTCGTTGAGGCAGAGAGTA	Construction of pAV43
**AV185**	CTGTTATGTGGTTATCGATTCCTAGCTTGTATGTCTGCGC	Construction of pAV43
**AV186**	AATCGATAACCACATAACAGTCATAAAAC	Construction of pAV43
**AV204**	ATAGGTATGTGGTTTTGTATTGGAAT	Construction of pAV43
**oGG38**	AAGATAAAGAATTTGCTCAAGACG	ϕNM4γ4α2 sequence verification
**oGG40**	ACCATTAAAACTCGTCATTCTTTC	ϕNM4γ4α2 sequence verification
**oGG297**	GCAAAATTCATATAAACAAATTAAAGAGGGTTATAATGAACG	Construction of pE194-*tyc1*
**oGG298**	CCCTCTTTAATTTGTTTATATGAATTTTGCTTATTAACGATTC	Construction of pE194-*tyc1*
**oGG315**	AGTGATCGTTAAATTTTAATCAAATATGTTCATAGCTTGATG	Construction of pGG90
**oGG316**	CGCATCCGATTGCAGCGATGCAGGTCTTTCTGTTGGC	Construction of pGG90
**oGG314**	AACATATTTGATTAAAATTTAACGATCACTCATCATGTTC	Construction of pGG90
**oGG317**	GAAAGACCTGCATCGCTGCAATCGGATGCGATTATTG	Construction of pGG90
**oGG192**	TCTACTTAATCTGATAAGTGAGC	ϕNM1-Erm^{R2} sequence verification
**oGG191**	GAAGCTTTAGCTTTGCAGTGG	ϕNM1-Erm^{R2} sequence verification
**oGG6**	TACCCTAGTTAACGTCTCTTG	ϕNM1 prophage genotyping
**oGG7**	GATATCAACTTGTAGTGCATCG	ϕNM1 prophage genotyping
**oGG10**	GCTGACTTACAAGAAGGTGGAC	ϕNM4 prophage genotyping
**oGG11**	GTTGTAATTGGATTAAATTCAGTC	ϕNM4 prophage genotyping
**JW809**	GTTGTACAAGGGTTACAATTCTTAATGC	Construction of pGG170 and pGG172
**JW810**	CCATCTTCATCAGCTGACATTAGAG	Construction of pGG170 and pGG172
**oGG467**	AATAACTCTAATGTCAGCTGATGAAGATGGGTTTACGACCGTTAAAAGCAAATG	Construction of pGG170
**oGG468**	AGTTCTGATCAACGTACATCGTAATTAGAATACGATTGCAAG	Construction of pGG170
**oGG469**	TTCTAATTACGATGTACGTTGATCAGAACTTAACAAGGAGG	Construction of pGG170
**oGG470**	TTGCATTAAGAATTGTAACCCTTGTACAACGAAAACCCTTTAACTTTTGATGTTG	Construction of pGG170
**oGG471**	AATAACTCTAATGTCAGCTGATGAAGATGGTTCAACTACATATTTGTAGCCTTC	Construction of pGG172
**oGG472**	AATAAACGGAGATGCCATGAATAAAATACTCGCTAACGGTTC	Construction of pGG172
**oGG473**	GTATTTTATTCATGGCATCTCCGTTTATTTTTAGTGCTATAC	Construction of pGG172
**oGG474**	TTGCATTAAGAATTGTAACCCTTGTACAACGCTTTCAAAAATTCCATAAAAGAAATC	Construction of pGG172
**W1250**	TTCAAAGAGTTGGTAGCTCAGAG	pGG170/pGG172 homology arm sequencing
**oGG455**	CATACCCATTCTAGGTTCAGTTC	pGG170 homology arm sequencing
**W1245**	GACAAAAATCACCTTGCGCTAATGCTCTGTTACAGCTGTTAGATTATGAAAGCCGATG	Sequencing of the *repA*(ts) region
**W354**	GTTTTTCAAAATCTGCGGTTGCG	Sequencing of the *repA*(ts) region
**B266**	TGGAATAATAGTAATATTATACAAAATGGA	Sequencing of the *repA*(ts) region
**W653**	ATTTACCGCTATCTTTACAGGTAC	Sequencing of the *repA*(ts) region
**oGG509**	TGGAAAGAAAAATTAGAGAGTTTGGGCGTATCTATGGC	Construction of pGG170ts and pGG172ts
**oGG510**	TACGCCCAAACTCTCTAATTTTTCTTTCCAATCATTAGGAATTG	Construction of pGG170ts and pGG172ts
**oGG32**	GTATTTTAAGGGTTGCAATTACG	Verification of pGG172ts integrants
**oGG33**	GTATCTGTTTTCAACTACATATTTG	Sequencing of GWG7
**oGG475**	AATAACTCTAATGTCAGCTGATGAAGATGGCGTAGTAAATAGATGTGTAATAAATG	Sequencing of GWG9

Supplementary References

1 Marraffini, L. A. & Sontheimer, E. J. Self versus non-self discrimination during CRISPR RNA-directed immunity. *Nature* **463**, 568-571 (2010).

2 Samai, P. *et al.* Co-transcriptional DNA and RNA Cleavage during Type III CRISPR-Cas Immunity. *Cell* **161**, 1164-1174, doi:10.1016/j.cell.2015.04.027 (2015).
